# Analysis of Mould Exposure of Immunosuppressed Patients at a German University Hospital

**DOI:** 10.3390/microorganisms11112652

**Published:** 2023-10-28

**Authors:** Danuta Puhlmann, Dominic Bergmann, Silke Besier, Michael Hogardt, Thomas A. Wichelhaus, Sabine Langhans, Daniel Hack, Claudia Reinheimer, Maria J. G. T. Vehreschild, Jens Jung, Volkhard A. J. Kempf

**Affiliations:** 1 Institute for Medical Microbiology and Infection Control, University Hospital, Goethe University Frankfurt, 60590 Frankfurt am Main, Germany; danuta.puhlmann@ukffm.de (D.P.);; 2 University Centre of Competence for Infection Control of the State of Hesse, 60590 Frankfurt am Main, Germany; 3 University Centre for Infectious Diseases (UCI), University Hospital, Goethe University Frankfurt, 60590 Frankfurt am Main, Germany; 4 Department for Internal Medicine II, University Hospital, Goethe University Frankfurt, 60590 Frankfurt am Main, Germany; 5 Department 1—Finance and Patient Services, University Hospital, 60590 Frankfurt am Main, Germany

**Keywords:** mould, immunosuppressed patients, microbial contamination, fungi, aspergillus, indoor air quality

## Abstract

Moulds are ubiquitous components of outdoor and indoor air and local conditions, temperature, humidity and season can influence their concentration in the air. The impact of these factors on mould exposure in hospitals and the resulting risk of infection for low to moderately immunocompromised patients is unclear. In the present retrospective analysis for the years 2018 to 2022, the monthly determined mould contamination of the outdoor and indoor air at the University Hospital Frankfurt am Main is compared with the average air temperature and the relative humidity. Mould infections (*Aspergillus* spp., Mucorales) of low to moderately immunosuppressed patients of a haematological-oncological normal ward were determined clinically according to the criteria of the European Organisation for Research and Treatment of Cancer (EORTC, Brussels, Belgium) and of the National Reference Centre for Surveillance of Nosocomial Infections (NRC-NI, Berlin, Germany). The data revealed that in the summer months (May–October), increased mould contamination was detectable in the outdoor and indoor air compared to the winter months (November–April). The mould levels in the patient rooms followed the detection rates of the outdoor air. Two nosocomial *Aspergillus* infections, one nosocomial Mucorales (*Rhizopus* spp.) infection (according to both NRC-NI and EORTC criteria) and five *Aspergillus* spp. infections (according to EORTC criteria) occurred in 4299 treated patients (resulting in 41,500 patient days). In our study, the incidence density rate of contracting a nosocomial mould infection (*n* = 3) was approximately 0.07 per 1000 patient days and appears to be negligible.

## 1. Introduction

Moulds occur naturally throughout nature and are a component of both outdoor and indoor air. Due to the small size of the spores (2–30 µm), they can travel long distances in the air. Humans are continuously exposed to mould spores [1,2,3,4,5]. Climate, time of day and year as well as local conditions influence the concentration of moulds in the air [1,4]. In nature, mould concentrations can range from 100 colony forming units (CFU) /m^3^ (in winter) to more than 2000 CFU/m^3^ (in summer) [1]. Other factors that influence the occurrence of moulds are temperature, humidity or the presence of rotting material [4].

Typical seasonal patterns of exposure to mould spores in outdoor air are known for certain mesophilic mould species such as *Cladosporium* sp., with the highest mould spore exposure occurring in the summer months (increasing from May to August, decreasing from August to October) [4,6]. Numerous moulds are known to differ significantly in their biological properties and preferred habitats. For example, *Aspergillus niger* is more commonly found in house dust or plant soil, while *Aspergillus fumigatus* tends to occur in low concentrations across the region in temperate climates, such as in Germany [4].

Human exposure to moulds can result in various clinical pictures ranging from allergic diseases to serious invasive infections caused by various thermotolerant mould species, the latter occurring mainly in at-risk groups (e.g., immunosuppressed patients) [1,7,8]. In immunocompromised patients, *A. fumigatus* can cause severe and often fatal lung infections (aspergillosis) [2,8,9,10]. In the case of clinical symptoms, mould infections in humans are detected by clinical microbiologically (by using, e.g., cultivation or molecular methods) and the diagnosis is supported by radiological imaging. Rapid detection of the *Aspergillus* galactomannan antigen in serum or bronchial secretions can significantly shorten the time to diagnose an aspergillosis [10,11,12,13].

Numerous national guidelines regulate the handling of mould contamination [4,7,14]. For many areas of health care and groups of people (except for severely or highly immunosuppressed patients), there are no strict recommendations for dealing with indoor air contaminated with mould, nor are there any limit values for mould concentrations [9]. This is also due to the fluctuating spore load in the outdoor air and the outdoor air parameters [1,4,9]. The interrelated assessment of mould contamination in outdoor areas and indoor spaces and its possible influence on mould infections (incl. invasive aspergillosis) are currently insufficiently described.

Based on routine airborne microbial measurements, we have created a comprehensive picture of outdoor air and indoor air pollution by thermotolerant moulds in a low-risk area (haematology/oncology normal ward) at the University Hospital Frankfurt am Main (UHF) between the years 2018 and 2022 and related these to nosocomial mould infections to derive possible infection prevention measures.

## 2. Materials and Methods

### 2.1. Ethics Vote

No ethics vote is required for the environmental analysis to detect mould contamination of outdoor and indoor air and for a retrospective and anonymised analysis of patient data (decision of the UHF Ethics Committee dated on 14 November 2018).

### 2.2. Airborne Microbial Measurement

Airborne microbial measurements were conducted monthly at the haematology/oncology ward (ward A0) between January 2018 and December 2022. A few specific measurements were not feasible in the period between 2019 and 2022 due to the COVID-19 pandemic and the resulting need to reorganise patient care. Ward A0 had to be temporarily relocated to other wards during the COVID-19 pandemic for organisational reasons (see Supplementary Figures S1–S3). The airborne microbial measurements as well as the associated laboratory tests were carried out under strict quality-assured conditions according to DIN EN ISO/IEC 17025 standards (certificate number D-PL-13102-01-00).

Airborne microbial measurements were carried out with a MAS 100 NT air sampler (MBV AG, Stäfa, Switzerland) in the centre of each patient room (without mechanical ventilation) and at three measuring points in the corridor area (see Supplementary Figures S1–S3). The external reference measuring points were located outside the respective hospital buildings (see Supplementary Figures S4–S6).

Airborne microbial measurement was carried out using the impaction method. For this, 250 l of air is aspirated in a time of 2 min and 30 s directly onto an agar-based medium plate (malt extract agar, Oxoid, Wesel, Germany) for mould growth. The culture medium plate was removed free of contamination, sealed airtight and then incubated at the Institute for Medical Microbiology and Infection Control for 48 h at 36 °C (incubator B60, Memmert, Schwabach, Germany). Microbial contamination was determined by counting the colonies using the so-called “Feller table” [15]. Laboratory data were documented in the hytec laboratory system (Epinet V.5.2023.01.07, Bochum, Germany). Cultivated moulds were identified using established routine methods (culture, microscopy, etc.); molecular or mass spectrometric analyses were not applied.

**Figure 1:**
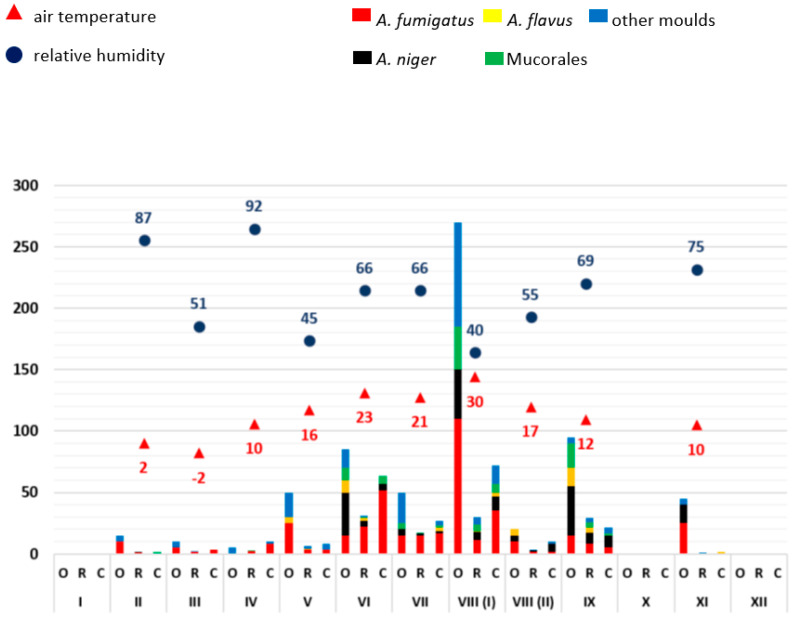
Overview of the frequency of mould detections at ward A0 in 2018. The moulds detected are colour-coded (see key in the figure). The average relative outdoor humidity in % and the air temperature in °C (2 m above the ground) are also shown. The months are indicated in Roman numerals. The number of pathogens detected is shown as colony-forming units (CFU/m^3^; mean values). O: measured values outside the building (reference measuring point), R: measured values in patient rooms, C: measured values in the corridor.

**Figure 2:**
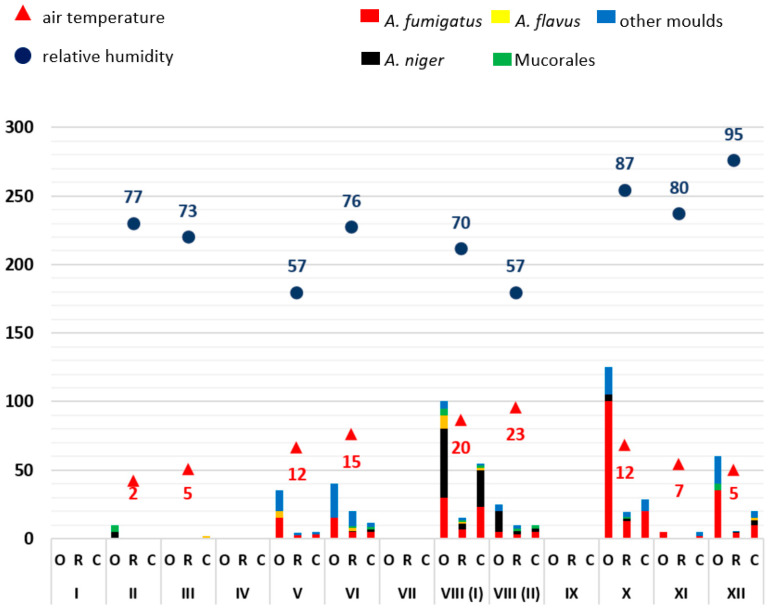
Overview of the frequency of mould detections at ward A0 in 2019. The moulds detected are colour-coded (see key in the figure). The average relative outdoor humidity in % and the air temperature in °C (2 m above the ground) are also shown. The months are indicated in Roman numerals. The number of pathogens detected is shown as colony-forming units (CFU/m^3^; mean values). O: measured values outside the building (reference measuring point), R: measured values in patient rooms, C: measured values in the corridor.

**Figure 3:**
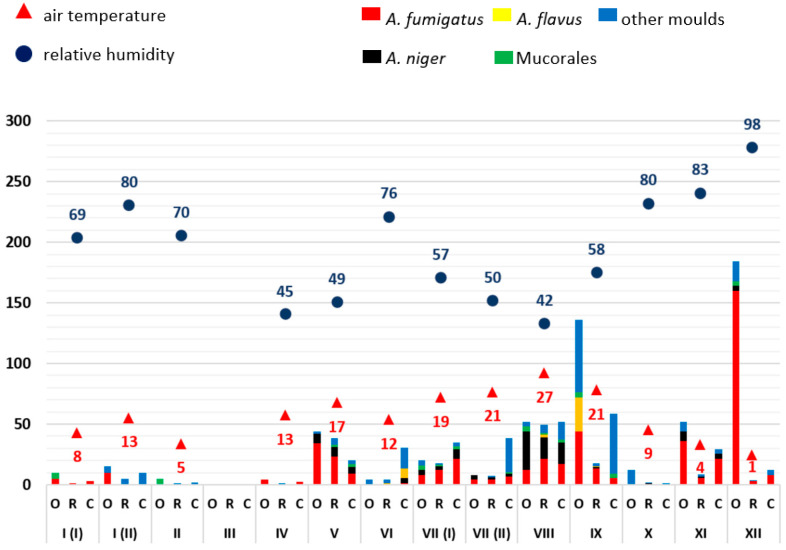
Overview of the frequency of mould detections at ward A0 (with relocation) in 2020. The moulds detected are colour-coded (see key in the figure). The average relative outdoor humidity in % and the air temperature in °C (2 m above the ground) are also shown. The months are indicated in Roman numerals. The number of pathogens detected is shown as colony-forming units (CFU/m^3^; mean values). O: measured values outside the building (reference measuring point), R: measured values in patient rooms, C: measured values in the corridor.

**Figure 4:**
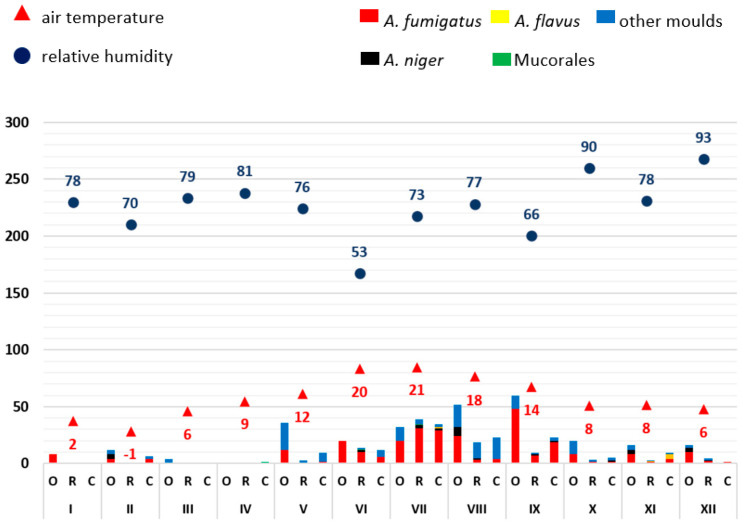
Overview of the frequency of mould detections at ward A0 (with relocation) in 2021. The moulds detected are colour-coded (see key in the figure). The average relative outdoor humidity in % and the air temperature in °C (2 m above the ground) are also shown. The months are indicated in Roman numerals. The number of pathogens detected is shown as colony-forming units (CFU/m^3^; mean values). O: measured values outside the building (reference measuring point), R: measured values in patient rooms, C: measured values in the corridor.

**Figure 5:**
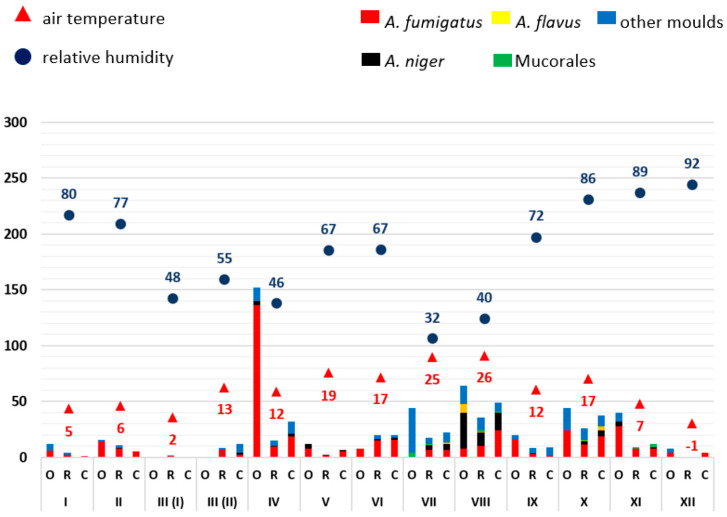
Overview of the frequency of mould detections at ward A0 (with relocation) in 2022. The moulds detected are colour-coded (see key in the figure). The average relative outdoor humidity in % and the air temperature in °C (2 m above the ground) are also shown. The months are indicated in Roman numerals. The number of pathogens detected is shown as colony-forming units (CFU/m^3^; mean values). O: measured values outside the building (reference measuring point), R: measured values in patient rooms, C: measured values in the corridor.

From the available results of the airborne microbial measurements, the mean values (see Supplementary Tables S1–S5) of the outdoor reference readings (O), patient rooms (R) and corridors (C), each for one sample day, were calculated and displayed graphically (Excel, Microsoft, Redmond, WA, USA) (Figure 1, Figure 2, Figure 3, Figure 4 and Figure 5). The individual measured values are presented as original data in the supplement (see Supplementary Tables S6–S10).

### 2.3. Weather Data Collection

The mean air temperature and relative humidity (measured 2 m above the ground) of the outdoor air were determined retrospectively using the data collected by the Frankfurt am Main weather station (ID 1420) of the Climate Data Centre of the German Weather Service (Deutscher Wetterdienst, DWD) [16]. The individual measured values are presented as original data in the supplement (see Supplementary Tables S11–S15).

### 2.4. Detection of Aspergillus-Infections Using Galactomannan Antigen Detections

For the detection of *Aspergillus* infection, galactomannan as a cell wall component of *Aspergillus* spp. was analysed from sera and bronchioalveolar lavages of in-patients on ward A0 (from 1 January 2018 to 31 December 2022) with suspected *Aspergillus* infections [13,17], using the Platelia *Aspergillus* ELISA (BioRad, Dreieich, Germany) according to the manufacturer’s instructions.

### 2.5. Laboratory Testing of Patient Specimen

All laboratory testing was performed under strict quality-controlled DIN ISO 15189:2014 standards (certificate number D–ML–13102–01–00).

### 2.6. Fungal Culture

Microbiological diagnosis of invasive pulmonary aspergillosis (IPA) was performed by microscopy, fungal culture and PCR (see below) from respiratory samples (sputum, bronchial secretions or bronchoalveolar lavages). Microscopic detection of filamentous fungi was performed using calcofluor white staining. For routine fungal cultures, standard media (Sabouraud dextrose agar; all Oxoid, Wesel, Germany) were inoculated and incubated at least for 7 days (initially 2 days at 37 °C and subsequently 5 days at room temperature to cover the growth of environmental fungal species on the basis of diagnostic standards [18]). In case of suspected IPA, incubation time was prolonged up to 14 days (2 days at 37 °C and 12 days at 30 °C) and, additionally, Sabouraud dextrose broth (Oxoid, Wesel, Germany) was inoculated.

### 2.7. Laboratory Panfungal, Aspergillus and Mucorales PCR Assays

Molecular detection of moulds was performed from respiratory samples (sputum, brochioalveolar lavage). For DNA-extraction, the High Pure PCR Template Preparation Kit (Roche, Penzberg, Germany) or the QIAsymphony DSP Virus/Pathogen Mini Kit (Qiagen, Hilden, Germany) was used in combination with the QIAsymphony platform. DNA extracts were subjected either to a (i) broad-range (panfungal) PCR targeting the 28S rRNA gene (primer 10F: 5′-GACATGGGTTAGTCGATCCTA-3′ and 12R 5′-CCTTATCTACATTYTTCTATCAAC-3′; 35 cycles: denaturation 30 s at 95 °C, annealing 30 sec at 53 °C, elongation 60 s at 72 °C) using Taq DNA Polymerase (with W-1)-Kit (Invitrogen, Darmstadt, Germany) and ROTI-Mix PCR3 nucleotides (Roth, Germany) as previously described [19], to a (ii) *Aspergillus*-specific PCR using the *A. fumigatus* DNA detection Kit according to the manufacturer′s instructions (Axonlab, Polling, Austria) or to a (iii) Mucorales-specific qualitative PCR targeting the 18S rRNA gene (primer ZM1mo, 5′-TTACCRTGAGCAAATCAGARTG-3′ and ZM3mod, 5′-AATCYAAGAATTTCACCTCTAGCG-3′, probe p-ZM, 5′-6FAM-TYRR(G)G(G)B(A)T(T)T(G)T(A)TTT-BBQ-3′; 50 cycles: denaturation 15 sec at 95 °C, annealing and elongation 60 s at 60 °C each) using LightCycler TaqMan Master-Kit (Roche, Mannheim, Germany) as previously described [20].

### 2.8. Retrospective Analysis of Nosocomial Aspergillus Infections in Immunocompromised Patients

In order to classify the clinical microbiological laboratory diagnostics appropriately with regard to their clinical relevance, the recommendations of the current version of the “Consensus Definitions of Invasive Fungal Disease from the European Organisation for Research and Treatment of Cancer (EORTC) and the Mycoses Study Group Education and Research Consortium” were applied [21]. In addition, according to the definition of the National Reference Centre for Surveillance of Nosocomial Infections (NRC-NI Berlin, Germany), infections (fungal, bacterial) are considered nosocomial at the earliest when symptoms begin on the third day of hospitalisation [22]. Infections existing on the day of admission or manifesting up to the second day after admission are consequently considered as community-acquired infections. The occupancy data of the inpatients with a length of stay of at least three days on the haematological-oncological ward A0 between January 2018 and December 2022 were determined using the EDP hospital information system Orbis (Dedalus Healthcare System Group, Bonn, Germany). Clinical microbiology laboratory data were determined by means of the EDP laboratory information system swisslab (Nexus, Berlin, Germany) and the corresponding patients were checked in clinical information system Orbis with regard to clinical signs of mould infection (Table 1).

## 3. Results

### 3.1. Monthly Mould Load Depending on Temperature and Air Humidity

As mould infections in immunocompromised patients are primarily transmitted via the air, the exposure of indoor air to moulds in patient rooms was first analysed over time. Mould measurements on the ward corridors and in front of the building (so-called outdoor reference measuring points) served as comparative measurements. The moulds were identified by routine microbiological methods (*A. fumigatus*, *A. niger*, *A. flavus*, Mucorales, other moulds not further differentiated). A possible influence of weather conditions on mould load of the outdoor air was related to the average relative humidity and air temperature. Data evaluation was carried out in descriptive terms by synopsis of the data in integrated graphical analyses (Figure 1, Figure 2, Figure 3, Figure 4 and Figure 5).

As an example, the results for the year 2022 are given here in detail: In 2022, the outdoor mould concentration from January to December was less than 50 CFU/m^3^. There were exceptions in April (more than 150 CFU/m^3^) and in August (more than 60 CFU/m^3^). In the first half of the year, temperatures ranged from 2 °C to 19 °C, and relative humidity varied between 46% and 80%. In the second half of the year, the outdoor temperature ranged from 26 °C to −1 °C and the relative humidity rose from 32% to 92%. Compared to all other moulds, *A. fumigatus* was consistently detected in the outside air as well as in the patients’ rooms and corridors.

*A. niger* detections occurred throughout the year, with an increase in the month of August (between 12 CFU/m^3^ and 32 CFU/m^3^). *A. flavus* and Mucorales were more frequently detected at the end of the year (July to November). In December, there was high humidity (92%) with low outdoor temperature (−1 °C), accompanied by a very low mould load (<10 CFU/m^3^).

In the other years, the following specific anomalies emerged: In December of 2020 and April of 2022, there were increased detection rates, especially for *A. fumigatus*; in 2021, only very low mould detection rates were found throughout the year. The highest detection rate in this study was measured at the beginning of August 2018 with 270 CFU/m^3^, *A. fumigatus* with 110 CFU/m^3^ and other moulds with 85 CFU/m^3^. On this day, the average outdoor temperature was 30 °C and the relative humidity was 40%.

With regard to the number of moulds detected, a trend towards seasonal variation was identified over five years. In the summer months (May–October) there were increased detection rates in outdoor and indoor air compared to the winter months (November–April) with individual exceptions in December 2020 and April 2022. No clear relationship was established between outdoor temperature or humidity. In each of the years between 2018 and 2022, the months of December to April (except December 2019 and 2020) had the lowest mould concentrations in outdoor air. The mould load in the outside air usually exceeded the amount detected in the patient rooms and ward corridors.

The data show that no clear correlation can be established between humidity and temperature on the seasonal air load of moulds. The low detection rates in the months of November to April and the higher detection rates in the months of May to October are notable.

### 3.2. Analysis of the Frequency of Aspergillus Antigen Detection and Mould Infections

The next step was to identify mould infections in immunosuppressed haematological oncology patients on ward A0 over the observation period from 1 January 2018 to 31 December 2022 and to check a possible relation to season, temperature, humidity and mould contamination of outdoor and indoor air. For this purpose, the monthly number of all patients with a length of stay of at least three days on ward A0 as well as the patient days were determined. In addition, a systematic, retrospective query from the *swisslab* laboratory information system determined the clinical microbiology laboratory results which were performed during this period to detect mould infections (*Aspergillus* galactomannan antigen, culture and PCR-based detections). Moreover, the corresponding clinical patient data were retrospectively checked by using the *Orbis* hospital information system with regard to clinical signs of a patient′s mould infection.

In 2018, one (June 2018) clinically suspected nosocomial mould infection with *Aspergillus* spp. (antigen test) defined according to EORTC and NRC-NI criteria was detected for 1006 patients. Three further *Aspergillus* detections, which were to be assessed as community-acquired infections according to NRC-NI criteria (once with *Aspergillus* spp., antigen test, twice *A. fumigatus*, culture), did not fulfil the EORTC criteria of an infection. In 2019, one (November 2019) probable *Aspergillus niger* infection (culture) according to EORTC criteria was detected for 919 patients (community-acquired according to NRC-NI criteria); in addition, one other nosocomial Mucorales (*Rhizopus* spp., PCR) infection defined according to EORTC criteria as clinically probable and defined by NRC-NI criteria was detected. The further *Aspergillus niger* detection (culture), which was to be assessed as a community-acquired infection according to NRC-NI criteria, did not fulfil the EORTC criteria of an infection. In 2020, a clinically probable *Aspergillus* infection according to EORTC criteria and nosocomial *Aspergillus* spp. defined according to NRC-NI criteria was diagnosed by one patient [August 2020; *Aspergillus* spp., (PCR and antigen test), *A. flavus* and *A. fumigatus* (culture)] for 831 patients. In 2021, no *Aspergillus* infection was detected for 788 patients. The further *A. fumigatus* and *A. versicolor* detection (culture), which was to be assessed as a community-acquired infection according to NRC-NI criteria, did not fulfil the EORTC criteria of an infection. In 2022, no *Aspergillus* infections were detected for 755 patients according to NRC-NIZ criteria. Two patients were found to be infected with *Aspergillus* spp. by PCR and antigen test (August 2022: confirmed infection according to EORTC criteria, December 2022: probable infection according to EORTC criteria) (Table 1). The further *Aspergillus* spp (PCR and antigen test), *A. fumigatus* (culture) and *A. niger* detections (culture), which were to be assessed as a community-acquired infections according to NRC-NI criteria, did not fulfil the EORTC criteria.

From these data, it appears that there was rarely a clinical indication to perform a mould infection diagnosis (e.g., for 4299 patients, only 136 laboratory requests to perform a galactomannan test were available). Six out of 136 patients tested positive for galactomannan. In total, six mould infections were detected based on the available diagnostics (antigen test, PCR and culture). Three patients were found to have a nosocomial mould infection (*Aspergillus* spp., *Aspergillus fumigatus*, *Rhizopus* spp.). According to the EORTC criteria, 6 patients suffered from a mould infection (five times probable *Aspergillus* spp. infection, one confirmed *Aspergillus* spp. infection and one infection with *Rhizopus* spp). In relation to all patient days (*n* = 41,500), the incidence density rate for a nosocomial mould infection is calculated 0.07 per 1000 patient days. There was no clear evidence of a dependency of mould infections on temperature, humidity or mould contamination in the indoor and outdoor air. Both, nosocomial infections and infections according to EORTC criteria were mainly detected in the summer months (June to August) excluding the year 2019 with only one infection according to EORTC criteria.

## 4. Discussion

Moulds can cause severe infections in immunocompromised patients. In particular, patients with underlying haematological-oncological diseases represent a risk group for mould infections; such infections are associated with an unfavourable prognosis in this patient population [2,5,8,9,23]. In the present retrospective study at the University Hospital Frankfurt am Main, we recorded the mould load in indoor air on a haematological-oncological ward over a period of five years (2018–2022) and related this to the mould load in outdoor air. Furthermore, we recorded the clinically manifest mould infections in the corresponding haematological-oncological patients during this period. To our knowledge, our study is the study with the longest observation period in this regard. It is important to mention that his study cannot establish causative links between exposure and infections.

Moulds occur in the outdoor and indoor air and in different concentrations depending on the season [1,2,3,4]. As a consequence, patient groups with an increased risk of infection (e.g., highly immunosuppressed patients undergoing chemotherapy, stem cell transplantation) are cared for in rooms with a room air conditioning system and filtered air [9] ensuring the absence of moulds. Other, less immunosuppressed patients are often cared for in rooms without an air-conditioning system and are therefore exposed to the regular mould contamination of the air. In addition to the natural mould load, building measures (e.g., renovations) or moisture damage can increase the mould load in the air and thus the risk of infection for the immunocompromised patients [24,25].

Our evaluation of the contamination of outdoor and indoor air with moulds yielded three main results: (i) in the warmer months (May–October), increased mould levels were detectable in the outdoor air, (ii) mould levels in the air of patient rooms followed (at lower concentrations) the detection rates in the outdoor air, and (iii) *A. fumigatus* was the most frequently detected mould.

The basic mould contamination of the outdoor air varied between 0 and 270 CFU/m^3^ over the observation period. In the months April–May to September–October, higher mould concentrations were usually present in the outdoor air; in the months November to March, the detection rates were generally lower. These fluctuations seem to be most likely due to an increased ambient temperature, a connection with humidity is not obvious. Individual fluctuations in mould levels (e.g., in December 2020) cannot be explained. Neither construction activities with excavated earth nor demolition works were in progress during this time. Retrospectively, it should be noted that the air measurements took place at different times during the day (usually between 8.00 am and 3.00 pm). The extent to which these different sampling times might have an influence on mould contamination (e.g., influence of traffic, thermal dissipation, etc.) would have to be analysed in more detail, as has already been done in other studies [24]. The low baseline mould levels in 2021 (up to 65 CFU/m^3^ in October 2021 compared to 100 and 300 CFU/m^3^ in August 2018 or October 2019) are striking. The reason for this may have been the reduced air circulation due to reduced road traffic during the COVID-19 pandemic.

The contamination of the air with mould spores in the patient rooms always followed the detection rates of mould spores in the outdoor air. However, detection rates in the patient rooms were generally lower than in the outside air. Causes for this can be the ventilation behaviour of the rooms (e.g., increased airing in summer) [24]. In addition, the patient rooms are located in the direction of an inner courtyard, which is different in terms of air circulation from the outdoor reference measuring points the latter located in the direction of a large flowing watercourse (the Main). It is important to mention that we analysed mould levels monthly in our retrospective study. A more frequent sampling would probably make it possible to reduce the variability in mould levels, however, this would be associated with workload in samples drawing and subsequent laboratory analysis.

It remains to be analysed whether elevated mould concentrations (e.g., of up to 100 CFU/m^3^) can be tolerated in terms of patient safety if, for example, the indoor concentration does not permanently exceed the outdoor concentration and a single mould species is not consistently present in elevated concentrations [24,26]. Concentrations of >50 CFU/m^3^ of a single mould species, based on indoor mean values (e.g., patient rooms), which might indicate increased exposure [26], were not reached in this study. Contrary to the assertion that soil fungi such as *A. fumigatus* are present in the air only in low concentrations (5–10 CFU/m^3^) [1], in our study *A. fumigatus* was present in higher concentrations throughout the year. This proves that limit values for mould contamination of outdoor air cannot be defined due to the high fluctuation range and are subject to regional influences.

In the entire observation period over five years, a total of only five *Aspergillus* infections and one Mucorales (*Rhizopus* spp.) infection were detected, with only three infections being formally (>48 h after patient admission) assessed as nosocomial infections. In one of the infections formally classified as nosocomial (June 2018), *Aspergillus* antigen detection from the patient’s serum sample remained negative within the first three days; after ten days, *Aspergillus* antigen was detected in this patient’s bronchial lavage. Based on the typical radiological findings (computed tomography of the thorax), a diagnosis of *Aspergillus* pneumonia was made according to EORTC criteria and the patient was treated with Voriconazole. Airborne microbial measurement on 8 June 2018 revealed increased outdoor air exposure (>80 CFU/m^3^), primarily with *A. niger* (35 CFU/m^3^) and simultaneously increased indoor air exposure with *A. fumigatus* in the patient room (22 CFU/m^3^; compared to outdoor air exposure: 15 CFU/m^3^). A causal relationship with the mould exposure in the patient’s room cannot be ruled out, as the patient had previously been treated in an inpatient area with an air-conditioning system and was considered severely immunosuppressed before that. There were five days between the admission to the haematological-oncological normal ward, before which, however, it can be assumed that the patient had been exposed to moulds in the private environment. In this respect, the diagnosis “nosocomial *Aspergillus* infection” is formally correct, but not provable due to the long incubation periods [27].

The other *Aspergillus* infection formally classified as nosocomial according to NRC-NI criteria was diagnosed via antigen test, PCR and culture in August 2020 in a patient 13 days after admission to the ward (31 August 2020). In addition to the laboratory findings, lung infiltrates were also detected on computed tomography and a diagnosis of *Aspergillus* infection was made and treated with Voriconazole. Airborne microbial measurements on 7 August revealed an outdoor air load of 52 CFU/m^3^ (*A. fumigatus* 12 CFU/m^3^, *A. niger* 32 CFU/m^3^). Elevated concentrations of *A. fumigatus* (21 CFU/m^3^) and *A. niger* (18 CFU/m^3^) were also present in the patient rooms. Ultimately, however, no provable connection between air exposure and infection is possible here either, given that the patient had been exposed in a private environment for a period of two days immediately beforehand. Generally, it is important to mention that such an analysis cannot prove causative links between mould exposure and infection. Our data definitively cannot establish causative links between exposure and infection. A determination of causative links would only be possible if both the respective environmental and patient mould isolates would have undergone, e.g., a molecular fine-typing. Such analyses would have great potential in determining the sources of (clonal) mould infections and should be analysed in future studies.

A general classification of *Aspergillus* infections as “community-acquired” or “nosocomial-acquired” seems problematic. In view of this difficulty, the clinical assessment “mould infection” was analysed again in all patients with laboratory evidence of moulds (antigen test, PCR, culture) in a reviewing manner according to EORTC criteria. Again, no temporal accumulation or cluster of mould infections was detectable. Interestingly, the infections formally classified as “nosocomial” as well as infections according to EORTC criteria were mainly detected in the summer months (June to August). Whether this phenomenon is due to the increased mould contamination of the air in these months remains questionable and should be further analysed in subsequent studies with a longer observation period.

## 5. Conclusions and Future Perspectives

From the above data, the two nosocomially defined *Aspergillus* infections in 4299 patients and 41,500 patient days result in an incidence density rate of 0.05 per 1000 patient days and one nosocomially defined Mucorales (*Rhizopus* spp.) infection results in an incidence density rate of 0.02 per 1000 patient days, which we consider to be a very low, possibly even negligible, risk. Due to this low risk, the lack of fluctuation in the detections over five years and the strongly fluctuating detection rates of the pathogens in outdoor and indoor air, it must be discussed whether a systematic recording of mould contamination makes sense. Moreover, in the case of increased detection rates in outdoor and, subsequently, in indoor air and the lack of available options for action, there is no consequence in infection control. Whether a defined mould concentration in the room air can be considered hazardous to the health of low to moderately immunocompromised patients is unlikely when viewed against the findings of our study. Precise limit values for mould contamination probably cannot be established due to the highly fluctuating detection rates and the negligible number of possible nosocomial infections with unclear reference to mould contamination in the room air on normal wards.

## Figures and Tables

**Table 1:** Overview of occupancy rates, diagnostics and mould infections, ward A0, years 2018–2022.

	**2018**												
**Month**	**01**	**02**	**03**	**04**	**05**	**06**	**07**	**08**	**09**	**10**	**11**	**12**	**Total**
**Patients ^a^ (n)**	87	74	99	84	86	75	93	85	79	88	79	77	**1006**
**Patient days ^a^ (n)**	715	631	804	680	739	756	852	732	725	867	841	676	**9018**
***Aspergillus* AG detection (n)**	0	0	0	0	0	**1**	0	0	0	0	0	**1**	**2**
***Aspergillus* culture pos. (n)**	0	**1**	0	**1**	0	0	0	0	0	0	0	0	**2**
***Aspergillus* PCR pos. (n)**	0	0	0	0	0	0	0	0	0	0	0	0	**0**
**Mucorales PCR pos. (n)**	0	0	0	0	0	0	0	0	0	0	0	0	**0**
**Mould infections NI ^b^ (n)**	0	0	0	0	0	**1**	0	0	0	0	0	0	**1**
**Mould infections EORTC (n) ^c^**	0	0	0	0	0	**1 ^d^**	0	0	0	0	0	0	**1**
	**2019**												
**Month**	**01**	**02**	**03**	**04**	**05**	**06**	**07**	**08**	**09**	**10**	**11**	**12**	**Total**
**Patients ^a^ (n)**	70	71	76	77	74	76	75	79	77	77	86	81	**919**
**Patient days ^a^ (n)**	724	735	794	810	789	743	735	852	885	713	844	729	**9353**
***Aspergillus* AG detection (n)**	0	0	0	0	0	0	0	0	0	0	0	0	**0**
***Aspergillus* culture pos. (n)**	0	0	0	0	0	0	0	0	**1**	0	**1**	0	**2**
***Aspergillus* PCR pos. (n)**	0	0	0	0	0	0	0	0	0	0	0	0	**0**
**Mucorales PCR pos. (n)**	0	0	0	0	0	0	**1**	0	0	0	0	0	**1**
**Mould infections NI ^b^ (n)**	0	0	0	0	0	0	**1**	0	0	0	0	0	**1**
**Mould infections EORTC (n) ^c^**	0	0	0	0	0	0	**1 ^d^**	0	0	0	**1 ^d^**	0	**2**
	**2020**												
**Month**	**01**	**02**	**03**	**04 ^e^**	**05 ^e^**	**06**	**07**	**08**	**09**	**10**	**11**	**12**	**Total**
**Patients ^a^ (n)**	77	68	87	70	74	77	81	62	60	69	50	56	**831**
**Patient days ^a^ (n)**	789	744	800	642	663	689	777	764	698	790	414	567	**8337**
***Aspergillus* AG detection (n)**	0	0	0	0	0	0	0	**1**	0	0	0	0	**1**
***Aspergillus* culture pos. (n)**	0	0	0	0	0	0	0	**1**	0	0	0	0	**1**
***Aspergillus* PCR pos. (n)**	0	0	0	0	0	0	0	**1**	0	0	0	0	**1**
**Mucorales PCR pos. (n)**	0	0	0	0	0	0	0	0	0	0	0	0	**0**
**Mould infections NI ^b^ (n)**	0	0	0	0	0	0	0	**1**	0	0	0	0	**1**
**Mould infections EORTC (n) ^c^**	0	0	0	0	0	0	0	**1 ^d^**	0	0	0	0	**1**
	**2021**												
**Month**	**01**	**02**	**03**	**04**	**05**	**06**	**07**	**08**	**09**	**10**	**11**	**12 ^f^**	**Total**
**Patients ^a^ (n)**	56	52	70	64	61	75	74	79	80	88	49	40	**788**
**Patient days ^a^ (n)**	590	532	686	616	613	792	693	792	861	841	508	269	**7793**
***Aspergillus* AG detection (n)**	0	0	0	0	0	0	0	0	0	0	0	0	**0**
***Aspergillus* culture pos. (n)**	0	0	**1**	0	0	0	0	0	**1**	0	0	0	**2**
***Aspergillus* PCR pos. (n)**	0	0	0	0	0	0	0	0	0	0	0	0	**0**
**Mucorales PCR pos. (n)**	0	0	0	0	0	0	0	0	0	0	0	0	**0**
**Mould infections NI ^b^ (n)**	0	0	0	0	0	0	0	0	0	0	0	0	**0**
**Mould infections EORTC (n) ^c^**	0	0	0	0	0	0	0	0	0	0	0	0	**0**
	**2022**												
**Month**	**01 ^f^**	**02 ^f^**	**03**	**04**	**05**	**06**	**07**	**08**	**09**	**10**	**11**	**12**	**Total**
**Patients ^a^ (n)**	56	45	37	46	59	75	76	59	71	71	80	80	**755**
**Patient days ^a^ (n)**	470	388	263	388	601	689	793	614	731	630	692	740	**6999**
***Aspergillus* AG detection (n)**	0	0	0	0	1	0	0	1	0	0	0	1	**3**
***Aspergillus* culture pos. (n)**	0	0	**1**	0	**2**	**1**	0	0	0	0	0	**1**	**5**
***Aspergillus* PCR pos. (n)**	0	0	0	0	0	0	0	**1**	0	0	0	**2**	**3**
**Mucorales PCR pos. (n)**	0	0	0	0	0	0	0	0	0	0	0	0	**0**
**Mould infections NI ^b^ (n)**	0	0	0	0	0	0	0	0	0	0	0	0	**0**
**Mould infections EORTC (n) ^c^**	0	0	0	0	0	0	0	**1 ^g^**	0	0	0	**1 ^d^**	**2**

^a^ Length of stay >3 days. ^b^ Nosocomial infection (NI) according to NRC-NI definition (>48h) [22]. ^c^ Infection according to EORTC criteria [21]. ^d^ Infection probable according to EORTC classification [21]. ^e^ Ward transfer from ward A0 (Building 23A) to ward 28-1 (Building 28). ^f^ Ward transfer from ward A0 (Building 23B) to ward 10B (Building 23B). ^g^ Infection confirmed according to EORTC classification [21].

## Data Availability

Not applicable.

## Supplementary Materials

The following supporting information can be downloaded at: https://www.mdpi.com/article/10.3390/microorganisms11112652/s1. Figure S1: Floor plan of ward A0, building 23A, ground floor (measuring points: blue dots), University Hospital Frankfurt (UHF), occupancy period January 2018 to March 2020; June 2020 to November 2021 and March 2022 to December 2022. Figure S2: Floor plan of ward 28-1 (A0), building 28, 1st floor (measuring points: blue dots), UHF, occupancy period April 2020 to May 2020. Figure S3: Floor plan of ward B10 (A0), building 23B, 10th upper floor (measuring points: blue dots), UHF, occupancy period December 2021 to February 2022. Figures S4, S5 and S6: External reference measuring points in front of House 23 and House 28 (measuring points: red dots), UHF. Table S1: Mean values of mould values (CFU/m^3^), 2018 ward A0, UHF. Table S2: Mean values of mould values (CFU/m^3^), 2019 ward A0, UHF. Table S3: Mean values of mould values (CFU/m^3^), 2020 ward A0, UHF. Table S4: Mean values of mould values (CFU/m^3^), 2021 ward A0, UHF. Table S5: Mean values of mould values (CFU/m^3^), 2022 ward A0, UHF. Table S6: Mould values (CFU/m^3^), raw data 2018, ward A0, UHF. Table S7: Mould values (CFU/m^3^), raw data 2019, ward A0, UHF. Table S8: Mould values (CFU/m^3^), raw data 2020, ward A0, UHF. Table S9: Mould values (CFU/m^3^), raw data 2021, ward A0, UHF. Table S10: Mould values (CFU/m^3^), raw data 2022, ward A0, UHF. Table S11: CDC weather data for weather station ID 1420 (2018). Table S12: CDC weather data for weather station ID 1420 (2019). Table S13: CDC weather data for weather station ID 1420 (2020). Table S14: CDC weather data for weather station ID 1420 (2021). Table S15: CDC weather data for weather station ID 1420 (2022).
