# Supplementary material for: Analysis of Mould Exposure of Immunosuppressed Patients at a German University Hospital
Source: Microorganisms. 2023 Oct 28;11(11):2652. doi: 10.3390/microorganisms11112652 (PMC10672964; doi:10.3390/microorganisms11112652)
Supplement: Supplementary file 1 [file microorganisms-11-02652-s001.zip › microorganisms-2628082-supplementary.pdf]

Supplementary Information

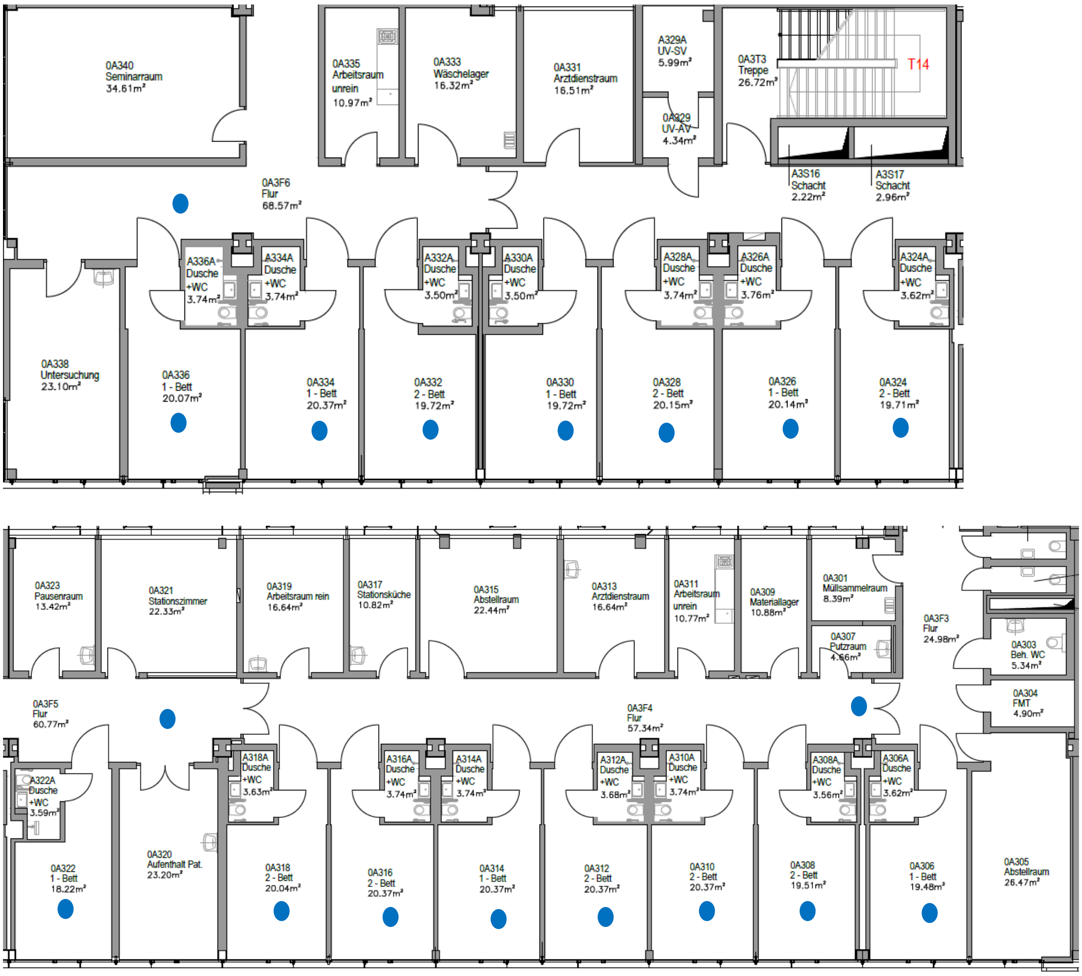

Figure S1: Floor plan of ward A0, building 23A, ground floor (measuring points: blue dots), University Hospital Frankfurt (UHF), occupancy period January 2018 to March 2020; June 2020 to November 2021 and March 2022 to December 2022.

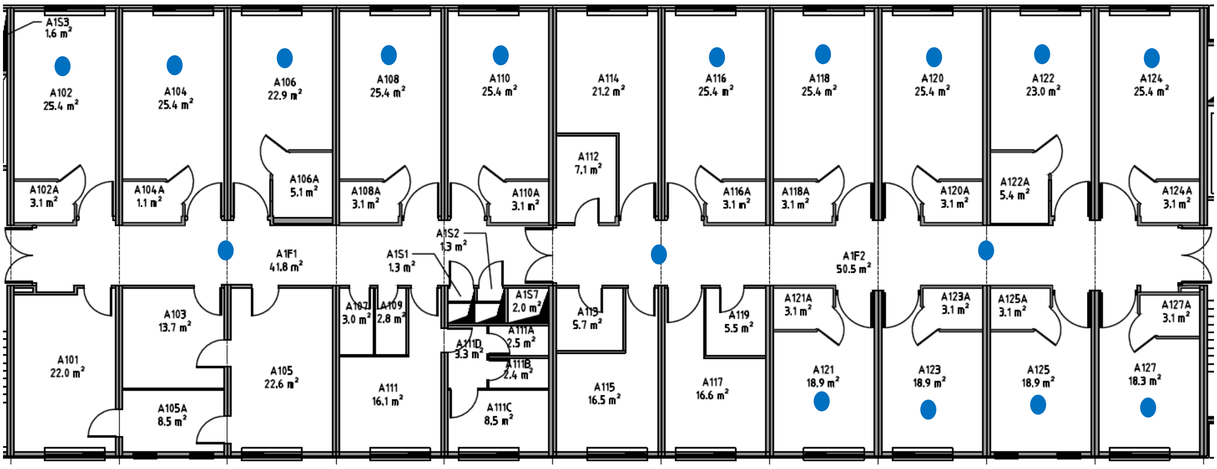

Figure S2: Floor plan of ward 28-1 (A0), building 28, 1st floor (measuring points: blue dots), UHF, occupancy period April 2020 to May 2020.

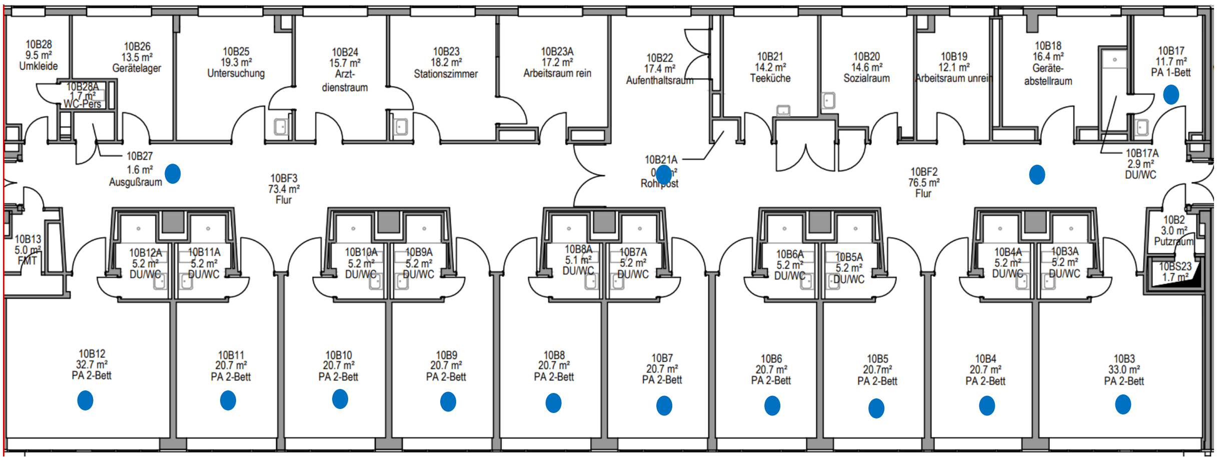

Figure S3: Floor plan of ward B10 (A0), building 23B, 10th upper floor (measuring points: blue dots), UHF, occupancy period December 2021 to February 2022.

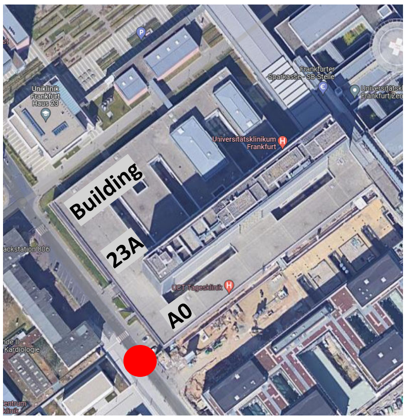

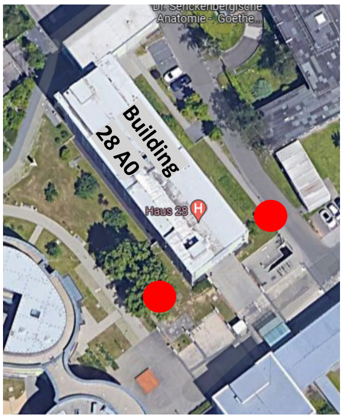

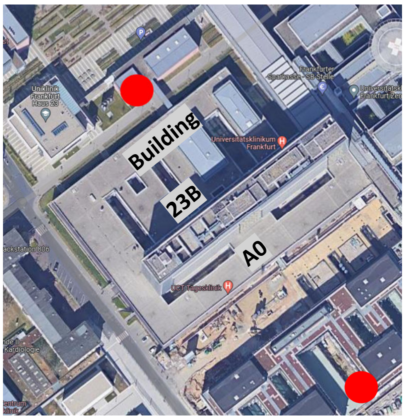

Figures S4, S5 and S6: External reference measuring points in front of House 23 and House 28 (measuring points: red dots), UHF.

Table S1: Mean values of mould values (CFU/m^3), 2018 ward A0, UHF.

2018/ Month	Location	*A. fumigatus*	*A. niger*	*A. flavus*	Mucorales	Other moulds
I	O	/	/	/	/	/
	R	/	/	/	/	/
	C	/	/	/	/	/
II	O	10	0	0	0	5
	R	1	0	0	0	0
	C	0	0	0	2	0
III	O	5	0	0	0	5
	R	2	0	0	0	0
	C	3	0	0	0	0
IV	O	0	0	0	0	5
	R	2	0	0	0	0
	C	8	0	0	0	2
V	O	25	0	5	0	20
	R	3	0	1	0	2
	C	3	0	0	0	5
VI	O	15	35	10	10	15
	R	22	5	2	1	1
	C	52	5	0	7	0
VII	O	15	5	0	5	25
	R	15	2	0	0	0
	C	17	2	3	2	3
VIII (I)	O	110	40	0	35	85
	R	11	7	1	5	6
	C	35	12	3	7	15
VIII (II)	O	10	5	5	0	0
	R	2	1	0	0	0
	C	2	7	0	0	2
IX	O	15	40	15	20	5
	R	8	9	4	4	3
	C	5	10	0	2	5
X	O	/	/	/	/	/
	R	/	/	/	/	/
	C	/	/	/	/	/
XI	O	25	15	0	0	5
	R	0	0	0	0	1
	C	0	0	2	0	0
XII	O	/	/	/	/	/
	R	/	/	/	/	/
	C	/	/	/	/	/

(I)beginning of the month; (II)end of the month; "/" = no measurement performed.

Table S2: Mean values of mould values (CFU/m^3), 2019 ward A0, UHF.

2019/ Month	Location	*A. fumigatus*	*A. niger*	*A. flavus*	Mucorales	Other moulds
I	O	/	/	/	/	/
	R	/	/	/	/	/
	C	/	/	/	/	/
II	O	0	5	0	5	0
	R	0	0	0	0	0
	C	0	0	0	0	0
III	O	0	0	0	0	0
	R	0	0	0	0	0
	C	0	0	2	0	0
IV	O	/	/	/	/	/
	R	/	/	/	/	/
	C	/	/	/	/	/
V	O	15	0	5	0	15
	R	2	0	0	0	2
	C	3	0	0	0	2
VI	O	15	0	0	0	25
	R	5	1	2	2	11
	C	5	2	0	2	3
VII	O	/	/	/	/	/
	R	/	/	/	/	/
	C	/	/	/	/	/
VIII (I)	O	30	50	10	5	5
	R	7	4	2	1	1
	C	23	27	2	2	2
VIII (II)	O	5	15	0	0	5
	R	3	3	0	1	3
	C	5	3	0	3	0
IX	O	/	/	/	/	/
	R	/	/	/	/	/
	C	/	/	/	/	/
X	O	100	5	0	0	20
	R	13	2	0	1	4
	C	20	0	0	0	8
XI	O	5	0	0	0	0
	R	0	0	0	0	0
	C	2	0	0	0	3
XII	O	35	0	0	5	20
	R	4	0	0	0	1
	C	10	3	2	0	5

(I)beginning of the month; (II)end of the month; "/" = no measurement performed.

Table S3: Mean values of mould values (CFU/m^3), 2020 ward A0, UHF.

2020/ Month	Location	*A. fumigatus*	*A. niger*	*A. flavus*	Mucorales	Other moulds
I (I)	O	5	0	0	5	0
	R	1	0	0	0	0
	C	3	0	0	0	0
I (II)	O	10	0	0	0	5
	R	0	0	0	0	5
	C	0	0	0	0	10
II	O	0	0	0	5	0
	R	0	0	0	0	1
	C	0	0	0	0	2
III	O	/	/	/	/	/
	R	/	/	/	/	/
	C	/	/	/	/	/
IV*	O	4	0	0	0	0
	R	1	0	0	0	1
	C	3	0	0	0	0
V*	O	34	8	0	0	2
	R	23	8	0	2	5
	C	9	5	0	3	3
VI	O	0	0	0	0	4
	R	1	0	1	0	3
	C	1	4	8	0	17
VII (I)	O	8	4	0	4	4
	R	12	3	0	1	1
	C	21	8	0	3	3
VII (II)	O	4	4	0	0	0
	R	5	1	0	0	1
	C	7	3	0	1	28
VIII	O	12	32	0	4	4
	R	21	18	2	1	7
	C	17	17	0	3	15
IX	O	44	0	28	4	60
	R	13	2	1	0	2
	C	5	0	0	4	49
X	O	0	0	0	0	12
	R	1	0	0	0	1
	C	0	0	0	0	1
XI	O	36	8	0	0	8
	R	6	1	0	0	2
	C	21	4	0	0	4
XII	O	160	4	0	4	16
	R	3	0	0	0	1
	C	8	0	0	0	4

(I)beginning of the month; (II)end of the month; * Ward relocation from Ward A0 (building 23A) to Ward 28-1 (building 28); "/" = no measurement performed.

Table S4: Mean values of mould values (CFU/m³), 2021 ward A0, UHF.

2021/ Month	Location	*A. fumigatus*	*A. niger*	*A. flavus*	Mucorales	Other moulds
I	O	8	0	0	0	0
	R	0	0	0	0	0
	C	0	0	0	0	0
II	O	4	4	0	0	4
	R	1	0	0	0	0
	C	4	0	0	0	3
III	O	0	0	0	0	4
	R	0	0	0	0	0
	C	0	0	0	0	0
IV	O	0	0	0	0	0
	R	1	0	0	0	0
	C	0	0	0	1	0
V	O	12	0	0	0	24
	R	0	0	0	0	3
	C	1	0	0	0	8
VI	O	20	0	0	0	0
	R	10	2	0	0	2
	C	6	0	0	0	6
VII	O	20	0	0	0	12
	R	31	3	0	1	4
	C	29	1	1	0	3
VIII	O	24	8	0	0	20
	R	3	1	0	0	14
	C	4	0	0	0	19
IX	O	48	0	0	0	12
	R	7	1	0	0	2
	C	19	1	0	0	3
X	O	8	0	0	0	12
	R	2	0	0	0	2
	C	1	1	0	0	3
XI	O	8	4	0	0	4
	R	1	0	1	0	1
	C	4	0	4	0	1
XII**	O	10	4	0	0	2
	R	2	1	0	0	2
	C	1	0	0	0	0

(I)beginning of the month; (II)end of the month; ** Ward relocation from Ward A0 (building 23A) to Ward 10B (building 23B).

Table S5: Mean values of mould values (CFU/m^3), 2022 ward A0, UHF.

2022/ Month	Location	*A. fumigatus*	*A. niger*	*A. flavus*	Mucorales	Other moulds
I**	O	6	0	0	0	6
	R	3	0	0	0	2
	C	1	0	0	0	0
II**	O	14	0	0	0	2
	R	8	1	0	0	2
	C	5	0	0	0	0
III (I)	O	0	0	0	0	0
	R	1	0	0	0	0
	C	0	0	0	0	0
III (II)	O	0	0	0	0	0
	R	6	0	0	0	2
	C	3	1	0	0	8
IV	O	136	4	0	0	12
	R	10	1	0	0	5
	C	19	3	0	0	11
V	O	8	4	0	0	0
	R	2	1	0	0	0
	C	5	1	0	0	0
VI	O	8	0	0	0	0
	R	15	1	0	0	3
	C	16	1	0	0	3
VII	O	0	0	0	4	40
	R	7	4	0	1	6
	C	7	5	1	0	9
VIII	O	8	32	8	0	16
	R	10	12	1	2	11
	C	24	16	0	1	8
IX	O	16	0	0	0	4
	R	3	1	0	0	5
	C	1	0	0	0	8
X	O	24	0	0	0	20
	R	11	3	1	1	10
	C	19	5	4	0	9
XI	O	28	4	0	0	8
	R	8	0	0	0	1
	C	8	1	0	3	0
XII	O	4	0	0	0	4
	R	0	0	0	0	0
	C	4	0	0	0	0

(I)beginning of the month; (II)end of the month; ** Ward relocation from Ward A0 (building 23A) to Ward 10B (building 23B).

Table S6: Mould values (CFU/m³), raw data 2018, ward A0, UHF.

**02.02.2018**	Sampling location	*A. fumigatus*	*A. niger*	*A. flavus*	Mucorales	Other moulds
	Room 306	10	2	0	0	0
	Room 308	0	0	0	0	0
	Room 310	0	0	0	0	0
	Room 312	0	0	0	0	0
	Room 314	0	0	0	0	0
	Room 316	0	0	0	0	0
	Room 318	0	0	0	0	0
	Room 322	0	0	0	0	0
	Room 324	0	0	0	0	0
	Room 326	5	0	0	0	0
	Room 328	0	0	0	0	0
	Room 330	0	0	0	0	0
	Room 332	0	0	0	0	0
	Room 334	0	0	0	0	0
	Room 336	0	0	0	0	0
	Front corridor	0	0	0	5	0
	Central corridor	0	0	0	0	0
	Rear corridor	0	0	0	0	0
	Reference measuring point next to building 23A in the direction of building 33/37	10	0	0	0	5
**02.03.2018**	Sampling location	*A. fumigatus*	*A. niger*	*A. flavus*	Mucorales	Other moulds
	Room 306	0	0	0	0	0
	Room 308	0	0	0	0	0
	Room 310	0	0	0	0	0
	Room 312	0	0	0	0	0
	Room 314	0	0	0	0	0
	Room 316	0	0	0	0	0
	Room 318	0	0	0	0	0
	Room 322	0	0	0	0	0
	Room 324	0	0	0	0	0
	Room 326	0	0	0	0	0
	Room 328	0	0	0	0	0
	Room 330	0	0	0	0	5
	Room 332	0	0	0	0	0
	Room 334	15	0	0	0	0
	Room 336	10	0	0	0	0
	Front corridor	5	0	0	0	0
	Central corridor	0	0	0	0	0
	Rear corridor	5	0	0	0	0
	Reference measuring point next to building 23A in the direction of building 33/37	5	0	0	0	5

**13.04.2018**	Sampling location	*A. fumigatus*	*A. niger*	*A. flavus*	Mucorales	Other moulds
	Room 306	0	0	0	0	0
	Room 308	0	0	0	0	0
	Room 310	5	0	0	0	0
	Room 312	5	0	0	0	0
	Room 314	0	0	0	0	0
	Room 316	5	0	0	0	0
	Room 318	0	0	0	0	0
	Room 322	10	0	0	0	0
	Room 324	5	0	0	0	0
	Room 326	0	0	0	0	0
	Room 328	0	0	0	0	0
	Room 330	0	0	0	5	0
	Room 332	0	0	0	0	0
	Room 334	0	0	0	0	0
	Room 336	0	0	0	0	0
	Front corridor	10	0	0	0	0
	Central corridor	15	0	0	0	0
	Rear corridor	0	0	0	0	5
	Reference measuring point next to building 23A in the direction of building 33/37	0	0	0	0	5

**04.05.2018**	Sampling location	*A. fumigatus*	*A. niger*	*A. flavus*	Mucorales	Other moulds
	Room 306	0	0	0	0	0
	Room 308	0	0	0	0	0
	Room 310	0	0	0	0	0
	Room 312	0	0	0	0	0
	Room 314	10	5	0	0	0
	Room 316	0	0	0	0	0
	Room 318	5	0	0	0	5
	Room 322	0	0	0	0	0
	Room 324	0	0	0	0	0
	Room 326	10	0	5	0	15
	Room 328	0	0	5	0	0
	Room 330	10	0	0	0	5
	Room 332	5	0	0	0	0
	Room 334	10	0	0	0	10
	Room 336	0	0	0	0	0
	Front corridor	5	0	0	0	0
	Central corridor	0	0	0	0	15
	Rear corridor	5	0	0	0	0
	Reference measuring point next to building 23A in the direction of building 33/37	25	0	5	0	20

**08.06.2018**	Sampling location	*A. fumigatus*	*A. niger*	*A. flavus*	Mucorales	Other moulds
	Room 306	30	0	5	0	0
	Room 308	25	0	5	0	0
	Room 310	25	0	0	0	0
	Room 312	40	0	10	0	0
	Room 314	10	10	0	0	0
	Room 316	50	10	0	0	0
	Room 318	10	10	0	0	0
	Room 322	10	0	10	0	0
	Room 324	20	0	0	0	0
	Room 326	15	20	0	0	10
	Room 328	50	15	0	0	10
	Room 330	45	10	0	0	0
	Room 332	0	0	0	10	0
	Room 334	0	0	0	0	0
	Room 336	0	0	0	0	0
	Front corridor	90	5	0	5	0
	Central corridor	40	10	0	10	0
	Rear corridor	25	0	0	5	0
	Reference measuring point next to building 23A in the direction of building 33/37	15	35	10	10	15
**05.07.2018**	Sampling location	*A. fumigatus*	*A. niger*	*A. flavus*	Mucorales	Other moulds
	Room 306	25	10	0	0	0
	Room 308	25	0	0	0	0
	Room 310	5	5	0	5	0
	Room 312	5	0	0	0	0
	Room 314	15	0	0	0	0
	Room 316	5	0	0	0	0
	Room 318	15	0	0	0	0
	Room 322	10	0	0	0	0
	Room 324	35	0	0	0	0
	Room 326	25	5	0	0	0
	Room 328	20	5	0	0	0
	Room 330	20	0	0	0	0
	Room 332	5	0	0	0	0
	Room 334	5	5	0	0	0
	Room 336	5	0	0	0	0
	Front corridor	20	5	5	0	5
	Central corridor	10	0	0	5	0
	Rear corridor	20	0	5	0	5
	Reference measuring point next to building 23A in the direction of building 33/37	15	5	0	5	25

**03.08.2018**	Sampling location	*A. fumigatus*	*A. niger*	*A. flavus*	Mucorales	Other moulds
	Room 306	25	35	0	10	20
	Room 308	10	0	0	0	0
	Room 310	15	0	0	5	5
	Room 312	10	10	0	5	5
	Room 314	10	0	0	5	0
	Room 316	10	5	5	10	5
	Room 318	20	40	0	10	10
	Room 322	10	0	0	15	15
	Room 324	5	0	0	0	5
	Room 326	0	0	0	0	0
	Room 328	0	0	0	0	0
	Room 330	10	10	5	10	10
	Room 332	15	0	0	0	10
	Room 334	10	0	0	0	5
	Room 336	15	5	0	5	0
	Front corridor	15	15	5	5	10
	Central corridor	50	5	5	10	20
	Rear corridor	40	15	0	5	15
	Reference measuring point next to building 23A in the direction of building 33/37	110	40	0	35	85
**31.08.2018**	Sampling location	*A. fumigatus*	*A. niger*	*A. flavus*	Mucorales	Other moulds
	Room 306	0	0	0	0	0
	Room 308	0	0	0	0	0
	Room 310	0	0	0	0	0
	Room 312	0	5	0	0	0
	Room 314	0	0	0	0	0
	Room 316	0	0	0	0	0
	Room 318	10	0	0	0	5
	Room 322	5	0	0	0	0
	Room 324	0	5	0	0	0
	Room 326	0	0	0	0	0
	Room 328	5	10	0	0	0
	Room 330	5	0	0	0	0
	Room 332	0	0	0	0	0
	Room 334	0	0	0	0	0
	Room 336	0	0	0	0	0
	Front corridor	0	15	0	0	0
	Central corridor	0	0	0	0	0
	Rear corridor	5	5	0	0	5
	Reference measuring point next to building 23A in the direction of building 33/37	10	5	5	0	0

**28.09.2018**	Sampling location	*A. fumigatus*	*A. niger*	*A. flavus*	Mucorales	Other moulds
	Room 306	0	0	0	0	0
	Room 308	5	15	0	0	10
	Room 310	0	5	0	5	5
	Room 312	0	10	0	10	0
	Room 314	10	10	0	5	5
	Room 316	5	0	0	5	0
	Room 318	20	10	10	10	5
	Room 322	10	35	20	10	15
	Room 324	20	5	5	0	0
	Room 326	20	20	10	5	5
	Room 328	0	0	0	0	0
	Room 330	5	0	5	0	0
	Room 332	15	5	5	5	5
	Room 334	5	15	10	5	0
	Room 336	5	10	0	0	0
	Front corridor	5	15	0	5	5
	Central corridor	5	10	0	0	5
	Rear corridor	5	5	0	0	5
	Reference measuring point next to building 23A in the direction of building 33/37	15	40	15	20	5
**02.11.2018**	Sampling location	*A. fumigatus*	*A. niger*	*A. flavus*	Mucorales	Other moulds
	Room 306	0	0	0	0	0
	Room 308	0	0	0	0	0
	Room 310	0	0	0	0	0
	Room 312	0	0	0	0	0
	Room 314	5	0	0	0	0
	Room 316	0	0	0	0	0
	Room 318	0	0	0	0	0
	Room 322	0	0	0	0	0
	Room 324	0	0	0	0	10
	Room 326	0	0	0	0	0
	Room 328	0	0	0	0	0
	Room 330	0	0	0	0	0
	Room 332	0	0	0	0	0
	Room 334	0	0	0	0	0
	Room 336	0	0	0	0	0
	Front corridor	0	0	5	0	0
	Central corridor	0	0	0	0	0
	Rear corridor	0	0	0	0	0
	Reference measuring point next to building 23A in the direction of building 33/37	25	15	0	0	5

Table S7: Mould values (CFU/m^3), raw data 2019, ward A0, UHF.

**05.02.2019**	Sampling location	*A. fumigatus*	*A. niger*	*A. flavus*	Mucorales	Other moulds
	Room 306	0	0	0	0	0
	Room 308	0	0	0	0	0
	Room 310	0	0	0	0	0
	Room 312	0	0	0	0	0
	Room 314	0	0	0	0	0
	Room 316	0	0	0	0	0
	Room 318	0	0	0	0	0
	Room 322	0	0	0	0	0
	Room 324	0	0	0	0	0
	Room 326	0	0	0	0	0
	Room 328	0	0	0	0	0
	Room 330	0	0	0	0	0
	Room 332	0	0	0	0	5
	Room 334	0	0	0	0	0
	Room 336	0	0	0	0	0
	Front corridor	0	0	0	0	0
	Central corridor	0	0	0	0	0
	Rear corridor	0	0	0	0	0
	Reference measuring point next to building 23A in the direction of building 33/37	0	5	0	5	0
**25.03.2019**	Sampling location	*A. fumigatus*	*A. niger*	*A. flavus*	Mucorales	Other moulds
	Room 306	0	0	0	0	0
	Room 308	0	0	0	0	0
	Room 310	0	0	0	0	0
	Room 312	0	0	0	0	0
	Room 314	0	0	0	0	0
	Room 316	0	0	0	0	0
	Room 318	0	0	0	0	0
	Room 322	0	0	0	0	0
	Room 324	5	0	0	0	0
	Room 326	0	0	0	0	0
	Room 328	0	0	0	0	0
	Room 330	0	0	0	0	0
	Room 332	0	0	0	0	0
	Room 334	0	0	0	0	0
	Room 336	0	0	0	0	0
	Front corridor	0	0	5	0	0
	Central corridor	0	0	0	0	0
	Rear corridor	0	0	0	0	0
	Reference measuring point next to building 23A in the direction of building 33/37	0	0	0	0	0

**03.05.2019**	Sampling location	*A. fumigatus*	*A. niger*	*A. flavus*	Mucorales	Other moulds
	Room 306	0	0	0	0	0
	Room 308	0	0	0	0	0
	Room 310	0	0	0	0	0
	Room 312	0	0	0	0	0
	Room 314	0	0	0	0	0
	Room 316	0	0	0	0	10
	Room 318	5	0	0	0	5
	Room 322	0	0	0	0	0
	Room 324	10	0	0	0	0
	Room 326	0	0	0	0	5
	Room 328	0	0	0	0	0
	Room 330	10	0	0	0	0
	Room 332	5	0	0	0	0
	Room 334	0	0	0	0	5
	Room 336	5	0	0	0	0
	Front corridor	5	0	0	0	0
	Central corridor	5	0	0	0	5
	Rear corridor	0	0	0	0	0
	Reference measuring point next to building 23A in the direction of building 33/37	15	0	5	0	15
**07.06.2019**	Sampling location	*A. fumigatus*	*A. niger*	*A. flavus*	Mucorales	Other moulds
	Room 306	0	0	35	0	80
	Room 308	0	0	0	0	0
	Room 310	0	0	0	0	0
	Room 312	10	5	0	0	0
	Room 314	10	0	0	0	10
	Room 316	0	0	0	5	15
	Room 318	10	0	0	0	10
	Room 322	0	0	0	0	0
	Room 324	0	0	0	0	5
	Room 326	10	5	0	10	0
	Room 328	5	0	0	5	0
	Room 330	0	0	0	0	25
	Room 332	15	0	0	5	0
	Room 334	10	0	0	0	10
	Room 336	0	0	0	0	10
	Front corridor	10	5	0	5	0
	Central corridor	5	0	0	0	5
	Rear corridor	0	0	0	0	5
	Reference measuring point next to building 23A in the direction of building 33/37	15	0	0	0	25

**02.08.2019**	Sampling location	*A. fumigatus*	*A. niger*	*A. flavus*	Mucorales	Other moulds
	Room 306	5	0	0	0	5
	Room 308	0	0	0	0	5
	Room 310	15	5	0	0	0
	Room 312	0	0	0	0	5
	Room 314	5	10	0	0	0
	Room 316	15	0	0	0	0
	Room 318	15	0	15	5	0
	Room 322	0	10	0	0	0
	Room 324	0	0	0	5	0
	Room 326	0	0	0	0	0
	Room 328	15	10	0	0	0
	Room 330	0	0	0	10	5
	Room 332	5	0	5	0	0
	Room 334	5	0	0	0	0
	Room 336	20	25	5	0	0
	Front corridor	0	20	0	0	0
	Central corridor	50	50	0	5	5
	Rear corridor	20	10	5	0	0
	Reference measuring point next to building 23A in the direction of building 33/37	30	50	10	5	5
**30.08.2019**	Sampling location	*A. fumigatus*	*A. niger*	*A. flavus*	Mucorales	Other moulds
	Room 306	0	0	0	0	5
	Room 308	0	0	0	0	0
	Room 310	0	0	0	0	5
	Room 312	0	0	0	5	0
	Room 314	10	5	0	0	5
	Room 316	10	10	0	0	5
	Room 318	0	0	0	5	0
	Room 322	0	10	0	0	0
	Room 324	0	10	0	5	5
	Room 326	0	0	0	0	10
	Room 328	5	5	0	0	0
	Room 332	/	/	/	/	/
	Room 334	5	0	0	0	5
	Room 336	10	0	0	5	0
	Front corridor	0	5	0	5	0
	Central corridor	10	0	0	0	0
	Rear corridor	/	/	/	/	/
	Reference measuring point next to building 23A in the direction of building 33/37	5	15	0	0	5

	Sampling location	*A. fumigatus*	*A. niger*	*A. flavus*	Mucorales	Other moulds
**11.10.2019**	Sampling location	*A. fumigatus*	*A. niger*	*A. flavus*	Mucorales	Other moulds
	Room 306	5	0	0	0	0
	Room 308	10	0	0	5	0
	Room 310	5	0	0	0	0
	Room 312	5	0	0	0	15
	Room 314	5	5	0	0	5
	Room 316	0	0	0	5	0
	Room 318	5	0	0	0	10
	Room 322	25	0	0	0	10
	Room 324	5	0	0	5	0
	Room 326	10	0	0	5	0
	Room 328	15	0	0	0	0
	Room 330	10	0	0	0	0
	Room 332	55	15	0	0	10
	Room 334	5	0	0	0	5
	Room 336	30	5	0	0	5
	Front corridor	10	0	0	0	0
	Central corridor	25	0	0	0	10
	Rear corridor	25	0	0	0	15
	Reference measuring point next to building 23A in the direction of building 33/37	100	5	0	0	20
**29.11.2019**	Sampling location	*A. fumigatus*	*A. niger*	*A. flavus*	Mucorales	Other moulds
	Room 306	5	0	0	0	0
	Room 308	0	0	0	0	0
	Room 310	0	0	0	0	0
	Room 312	0	0	0	0	0
	Room 314	0	0	0	0	0
	Room 316	0	0	0	0	0
	Room 318	0	0	0	0	0
	Room 322	0	0	0	0	0
	Room 324	0	0	0	0	0
	Room 326	0	0	0	0	0
	Room 328	0	0	0	0	0
	Room 330	0	0	0	0	0
	Room 332	0	0	0	0	0
	Room 334	0	0	0	0	0
	Room 336	0	0	0	0	0
	Front corridor	5	0	0	0	5
	Central corridor	0	0	0	0	0
	Rear corridor	0	0	0	0	5
	Reference measuring point next to building 23A in the direction of building 33/37	5	0	0	0	0

**19.12.2019**	Sampling location	*A. fumigatus*	*A. niger*	*A. flavus*	Mucorales	Other moulds
	Room 306	0	0	0	0	0
	Room 308	0	0	0	0	0
	Room 310	35	0	0	0	0
	Room 312	5	5	0	0	0
	Room 314	0	0	0	0	0
	Room 316	0	0	0	0	0
	Room 318	0	0	0	0	0
	Room 322	20	0	0	0	0
	Room 324	0	0	0	0	5
	Room 326	5	0	0	0	0
	Room 328	0	0	0	0	0
	Room 330	0	0	0	0	0
	Room 332	0	0	0	0	5
	Room 334	0	0	0	0	0
	Room 336	0	0	0	0	0
	Front corridor	25	0	5	0	15
	Central corridor	0	5	0	0	0
	Rear corridor	5	5	0	0	0
	Reference measuring point next to building 23A in the direction of building 33/37	35	0	0	5	20

Table S8: Mould values (CFU/m³), raw data 2020, ward A0, UHF.

	Sampling location	*A. fumigatus*	*A. niger*	*A. flavus*	Mucorales	Other moulds
**14.01.2020**	Room 306	0	0	0	0	0
	Room 308	0	0	0	0	0
	Room 310	0	0	0	0	0
	Room 312	0	0	0	0	0
	Room 314	0	0	0	0	0
	Room 316	0	0	0	0	0
	Room 318	0	0	0	0	0
	Room 322	5	0	0	0	0
	Room 324	0	0	0	0	0
	Room 326	0	0	0	0	0
	Room 328	0	0	0	0	0
	Room 330	0	0	0	0	0
	Room 332	0	0	0	0	0
	Room 334	10	0	0	0	0
	Room 336	5	0	0	0	0
	Front corridor	10	0	0	0	0
	Central corridor	0	0	0	0	0
	Rear corridor	0	0	0	0	0
	Reference measuring point next to building 23A in the direction of building 33/37	5	0	0	5	0
**31.01.2020**	Sampling location	*A. fumigatus*	*A. niger*	*A. flavus*	Mucorales	Other moulds
	Room 306	0	0	0	0	5
	Room 308	0	0	0	0	20
	Room 310	0	0	0	0	0
	Room 312	0	0	0	0	0
	Room 314	0	0	0	0	0
	Room 316	0	0	0	0	15
	Room 318	0	0	0	0	5
	Room 322	0	0	0	0	5
	Room 324	0	0	0	0	5
	Room 326	0	0	0	0	0
	Room 328	0	0	0	0	0
	Room 330	0	0	0	0	5
	Room 332	0	0	0	0	0
	Room 334	0	0	0	0	5
	Room 336	0	0	5	0	5
	Front corridor	0	0	0	0	25
	Central corridor	0	0	0	0	5
	Rear corridor	0	0	0	0	0
	Reference measuring point next to building 23A in the direction of building 33/37	10	0	0	0	5

**28.02.2020**	Sampling location	*A. fumigatus*	*A. niger*	*A. flavus*	Mucorales	Other moulds
	Room 306	0	0	0	0	0
	Room 308	0	0	0	0	0
	Room 310	0	0	0	0	0
	Room 312	0	0	0	0	0
	Room 314	0	0	0	0	0
	Room 316	0	0	0	0	0
	Room 318	0	0	0	0	0
	Room 322	0	0	0	0	0
	Room 324	0	0	0	0	0
	Room 326	0	0	0	0	0
	Room 328	0	0	0	0	0
	Room 330	0	0	0	0	5
	Room 332	0	0	0	0	0
	Room 334	5	0	0	0	0
	Room 336	0	5	0	0	5
	Front corridor	0	0	0	0	0
	Central corridor	0	0	0	0	5
	Rear corridor	0	0	0	0	0
	Reference measuring point next to building 23A in the direction of building 33/37	0	0	0	5	0
**06.04.2020***	Sampling location	*A. fumigatus*	*A. niger*	*A. flavus*	Mucorales	Other moulds
	Room A102H	0	0	0	0	4
	Room A104H	0	0	0	0	0
	Room A106H	0	0	0	0	0
	Room A108H	/	/	/	/	/
	Room A110H	0	0	0	0	4
	Room A116H	0	0	0	0	0
	Room A118H	0	0	0	0	0
	Room A120H	0	0	0	0	0
	Room A121H	4	0	0	0	0
	Room A122H	4	0	0	0	0
	Room A123H	0	0	0	0	0
	Room A124H	0	0	0	0	0
	Room A125H	0	0	0	0	0
	Front corridor	0	0	0	0	0
	Central corridor	8	0	0	0	0
	Rear corridor	0	0	0	0	0
	Reference measuring point next to building 28, staircase left, in front of the entrance	0	0	0	0	0
	Reference measuring point next to building 28, staircase on the right, in front of the entrance	8	0	0	0	0

**08.05.2020***	Sampling location	*A. fumigatus*	*A. niger*	*A. flavus*	Mucorales	Other moulds
	Room A102H	28	8	0	0	0
	Room A104H	28	12	0	0	0
	Room A106H	24	8	0	0	4
	Room A108H	20	12	0	4	8
	Room A110H	20	4	4	0	0
	Room A116H	28	4	0	4	8
	Room A118H	48	24	0	0	8
	Room A120H	36	8	0	4	16
	Room A121H	0	0	0	4	12
	Room A122H	56	12	0	0	16
	Room A123H	8	0	0	0	0
	Room A124H	8	8	0	4	4
	Room A125H	4	0	0	4	0
	Room A127H	16	8	0	0	0
	Front corridor	8	0	0	0	0
	Central corridor	16	8	0	4	0
	Rear corridor	4	8	0	4	8
	Reference measuring point next to building 28, staircase left, in front of the entrance	36	0	0	0	0
	Reference measuring point next to building 28, staircase on the right, in front of the entrance	32	16	0	0	4
**05.06.2020**	Sampling location	*A. fumigatus*	*A. niger*	*A. flavus*	Mucorales	Other moulds
	Room 306	0	0	0	0	0
	Room 308	4	0	0	0	16
	Room 310	0	0	0	0	8
	Room 312	0	0	0	0	4
	Room 314	0	0	0	0	0
	Room 316	4	0	0	0	0
	Room 318	0	0	0	0	0
	Room 322	0	0	0	0	4
	Room 324	0	0	0	0	4
	Room 326	0	0	0	0	8
	Room 328	0	0	0	0	0
	Room 330	0	0	0	0	0
	Room 332	0	0	0	0	0
	Room 334	4	0	0	0	0
	Room 336	0	0	8	0	0
	Front corridor	0	0	0	0	28
	Central corridor	0	0	0	0	24
	Rear corridor	4	12	24	0	0
	Reference measuring point next to building 23A in the direction of building 33/37	0	0	0	0	4

**03.07.2020**	Sampling location	*A. fumigatus*	*A. niger*	*A. flavus*	Mucorales	Other moulds
	Room 306	0	0	0	0	0
	Room 308	0	0	0	4	0
	Room 310	4	0	0	0	0
	Room 312	0	0	0	4	0
	Room 314	0	0	0	0	0
	Room 316	20	4	0	0	8
	Room 318	24	4	0	0	8
	Room 322	0	0	0	4	0
	Room 324	20	4	0	0	0
	Room 326	24	16	0	0	0
	Room 328	28	8	0	0	0
	Room 330	16	0	0	0	0
	Room 332	12	0	0	8	4
	Room 334	24	4	0	0	0
	Room 336	8	8	0	0	0
	Front corridor	4	0	0	4	4
	Central corridor	48	24	0	4	4
	Rear corridor	12	0	0	0	0
	Reference measuring point next to building 23A in the direction of building 33/37	8	4	0	4	4
**14.07.2020**	Sampling location	*A. fumigatus*	*A. niger*	*A. flavus*	Mucorales	Other moulds
	Room 306	4	0	0	0	0
	Room 308	8	4	0	0	0
	Room 310	4	0	0	0	0
	Room 312	8	0	0	0	4
	Room 314	0	0	0	0	0
	Room 316	8	0	0	4	0
	Room 318	8	0	0	0	0
	Room 322	4	0	0	0	0
	Room 324	0	0	0	0	0
	Room 326	4	4	0	0	8
	Room 328	4	0	0	0	0
	Room 330	0	0	0	0	0
	Room 332	8	4	0	0	0
	Room 334	4	4	0	0	4
	Room 336	4	4	0	0	0
	Front corridor	4	4	0	4	0
	Central corridor	8	4	0	0	0
	Rear corridor	8	0	0	0	84
	Reference measuring point next to building 23A in the direction of building 33/37	4	4	0	0	0

**07.08.2020**	Sampling location	*A. fumigatus*	*A. niger*	*A. flavus*	Mucorales	Other moulds
	Room 306	32	48	0	0	8
	Room 308	24	16	0	4	4
	Room 310	36	32	4	0	4
	Room 312	32	16	4	0	8
	Room 314	36	48	28	0	0
	Room 316	24	24	0	4	0
	Room 318	16	16	0	4	20
	Room 322	24	0	0	4	20
	Room 324	16	24	0	0	4
	Room 326	16	8	0	0	4
	Room 328	4	12	0	0	4
	Room 330	4	4	0	4	4
	Room 332	28	20	0	0	4
	Room 334	4	0	0	0	8
	Room 336	20	4	0	0	8
	Front corridor	8	0	0	4	28
	Central corridor	28	44	0	4	8
	Rear corridor	16	8	0	0	8
	Reference measuring point next to building 23A in the direction of building 33/37	12	32	0	4	4

**04.09.2020**	Sampling location	*A. fumigatus*	*A. niger*	*A. flavus*	Mucorales	Other moulds
	Room 306	4	0	0	0	4
	Room 308	4	8	0	0	4
	Room 310	8	0	0	0	8
	Room 312	28	0	4	0	0
	Room 314	0	0	0	0	4
	Room 316	0	4	4	0	0
	Room 318	40	8	0	0	4
	Room 322	12	4	0	0	4
	Room 324	20	0	0	0	0
	Room 326	0	0	0	0	0
	Room 328	28	0	0	0	4
	Room 330	0	0	0	0	0
	Room 332	0	0	0	0	4
	Room 334	20	0	0	0	0
	Room 336	36	0	0	0	0
	Front corridor	4	0	0	4	60
	Central corridor	8	0	0	4	44
	Rear corridor	4	0	0	4	44
	Reference measuring point next to building 23A in the direction of building 33/37	44	0	28	4	60

**19.10.2020**	Sampling location	*A. fumigatus*	*A. niger*	*A. flavus*	Mucorales	Other moulds
	Room 306	8	4	0	0	8
	Room 308	0	0	0	0	0
	Room 310	0	0	0	0	0
	Room 312	4	0	0	0	4
	Room 314	0	0	0	0	0
	Room 316	0	0	0	0	0
	Room 318	0	0	0	0	0
	Room 322	0	0	0	0	0
	Room 324	0	0	0	0	0
	Room 326	0	0	0	0	0
	Room 328	0	0	0	0	0
	Room 330	0	0	0	0	0
	Room 332	0	0	0	0	0
	Room 334	0	0	0	0	0
	Room 336	0	0	0	0	0
	Front corridor	0	0	0	0	4
	Central corridor	0	0	0	0	0
	Rear corridor	0	0	0	0	0
	Reference measuring point next to building 23A in the direction of building 33/37	0	0	0	0	12
**05.11.2020**	Sampling location	*A. fumigatus*	*A. niger*	*A. flavus*	Mucorales	Other moulds
	Room 306	8	4	0	0	0
	Room 308	0	0	0	0	0
	Room 310	0	0	0	0	0
	Room 312	0	0	0	0	0
	Room 314	0	0	0	0	0
	Room 316	8	4	0	0	0
	Room 318	0	0	0	0	0
	Room 322	0	0	0	0	0
	Room 324	28	4	0	0	8
	Room 326	0	0	0	0	0
	Room 328	4	0	0	0	0
	Room 330	0	0	0	0	0
	Room 332	8	4	0	0	8
	Room 334	4	0	0	0	4
	Room 336	24	0	0	0	8
	Front corridor	12	0	0	0	4
	Central corridor	40	8	0	0	0
	Rear corridor	12	4	0	0	8
	Reference measuring point next to building 23A in the direction of building 33/37	36	8	0	0	8

**01.12.2020**	Sampling location	*A. fumigatus*	*A. niger*	*A. flavus*	Mucorales	Other moulds
	Room 306	4	0	0	0	0
	Room 308	12	0	0	0	4
	Room 310	0	0	0	0	0
	Room 312	0	0	0	0	0
	Room 314	4	0	0	0	0
	Room 316	0	0	0	0	0
	Room 318	0	0	0	0	0
	Room 322	4	0	0	0	0
	Room 324	20	0	0	0	4
	Room 326	4	0	0	0	0
	Room 328	0	0	0	0	0
	Room 330	0	0	0	0	0
	Room 332	0	0	0	0	0
	Room 334	0	0	0	0	0
	Room 336	0	0	0	0	0
	Front corridor	12	0	0	0	4
	Central corridor	12	0	0	0	4
	Rear corridor	0	0	0	0	4
	Reference measuring point next to building 23A in the direction of building 33/37	160	4	0	4	16

***** ward relocation from ward A0 (building 23A) to ward 28-1 (building 28)

Table S9: Mould values (CFU/m³), raw data 2021, ward A0, UHF.

**26.01.2021**	Sampling location	*A. fumigatus*	*A. niger*	*A. flavus*	Mucorales	Other moulds
	Room 306	0	0	0	0	0
	Room 308	0	0	0	0	0
	Room 310	0	0	0	0	0
	Room 312	0	0	0	0	0
	Room 314	4	0	0	0	0
	Room 316	0	0	0	0	0
	Room 318	0	0	0	0	0
	Room 322	0	0	0	0	0
	Room 324	0	0	0	0	0
	Room 326	0	0	0	0	0
	Room 328	0	0	0	0	0
	Room 330	0	0	0	0	0
	Room 332	0	0	0	0	0
	Room 334	0	0	0	0	0
	Room 336	0	0	0	0	0
	Front corridor	0	0	0	0	0
	Central corridor	0	0	0	0	0
	Rear corridor	0	0	0	0	0
	Reference measuring point next to building 23A in the direction of building 33/37	8	0	0	0	0
**15.02.2021**	Sampling location	*A. fumigatus*	*A. niger*	*A. flavus*	Mucorales	Other moulds
	Room 306	0	0	0	0	0
	Room 308	0	0	0	0	0
	Room 310	0	0	0	0	0
	Room 312	0	0	0	0	0
	Room 314	0	0	0	0	0
	Room 316	4	0	0	0	0
	Room 318	0	0	0	0	0
	Room 322	0	0	0	0	0
	Room 324	0	0	0	0	0
	Room 326	0	0	0	0	0
	Room 328	4	0	0	0	0
	Room 330	0	0	0	0	0
	Room 332	0	0	0	0	0
	Room 334	0	0	0	0	0
	Room 336	0	0	0	0	0
	Front corridor	0	0	0	0	0
	Central corridor	8	0	0	0	8
	Rear corridor	4	0	0	0	0
	Reference measuring point next to building 23A in the direction of building 33/37	4	4	0	0	4

**15.03.2021**	Sampling location	*A. fumigatus*	*A. niger*	*A. flavus*	Mucorales	Other moulds
	Room 306	0	0	0	0	0
	Room 308	0	0	0	0	0
	Room 310	0	0	0	0	0
	Room 312	0	0	0	0	0
	Room 314	0	0	0	0	0
	Room 316	0	0	0	0	0
	Room 318	0	0	0	0	0
	Room 322	0	0	0	0	0
	Room 324	0	0	0	0	0
	Room 326	0	0	0	0	0
	Room 328	0	0	0	0	0
	Room 330	0	0	0	0	0
	Room 332	0	0	0	0	0
	Room 334	0	0	0	0	0
	Room 336	0	0	0	0	0
	Front corridor	0	0	0	0	0
	Central corridor	0	0	0	0	0
	Rear corridor	0	0	0	0	0
	Reference measuring point next to building 23A in the direction of building 33/37	0	0	0	0	4
**19.04.2021**	Sampling location	*A. fumigatus*	*A. niger*	*A. flavus*	Mucorales	Other moulds
	Room 306	0	0	0	0	0
	Room 308	4	0	0	0	0
	Room 310	0	0	0	0	0
	Room 312	0	0	0	0	0
	Room 314	0	0	0	0	0
	Room 316	0	0	0	0	0
	Room 318	4	0	0	0	0
	Room 322	0	0	0	0	0
	Room 324	0	0	0	0	0
	Room 326	0	0	0	0	0
	Room 328	0	0	0	0	0
	Room 330	4	0	0	0	0
	Room 332	0	0	0	0	0
	Room 334	0	0	0	0	0
	Room 336	4	0	0	0	0
	Front corridor	0	0	0	0	0
	Central corridor	0	0	0	4	0
	Rear corridor	0	0	0	0	0
	Reference measuring point next to building 23A in the direction of building 33/37	0	0	0	0	0

	Sampling location	*A. fumigatus*	*A. niger*	*A. flavus*	Mucorales	Other moulds
**18.05.2021**	Sampling location	*A. fumigatus*	*A. niger*	*A. flavus*	Mucorales	Other moulds
	Room 306	0	0	0	0	0
	Room 308	0	0	0	0	16
	Room 310	0	0	0	0	0
	Room 312	0	0	0	0	4
	Room 314	0	0	0	0	4
	Room 316	0	0	0	0	4
	Room 318	0	0	0	0	0
	Room 322	0	0	0	0	0
	Room 324	0	0	0	0	0
	Room 326	0	0	0	0	4
	Room 328	0	0	0	0	0
	Room 330	0	0	0	0	0
	Room 332	0	0	0	0	0
	Room 334	0	0	0	0	4
	Room 336	0	0	0	0	4
	Front corridor	0	0	0	0	8
	Central corridor	0	0	0	0	12
	Rear corridor	4	0	0	0	4
	Reference measuring point next to building 23A in the direction of building 33/37	12	0	0	0	24
**14.06.2021**	Sampling location	*A. fumigatus*	*A. niger*	*A. flavus*	Mucorales	Other moulds
	Room 306	0	0	0	0	0
	Room 308	12	0	0	0	4
	Room 310	0	4	0	0	0
	Room 312	8	4	0	0	12
	Room 314	0	0	0	0	4
	Room 316	8	0	4	0	0
	Room 318	4	0	0	0	0
	Room 322	0	0	0	0	0
	Room 324	0	12	0	0	0
	Room 326	8	0	0	0	0
	Room 328	20	0	0	0	4
	Room 330	52	0	0	0	0
	Room 332	28	4	0	0	0
	Room 334	8	0	0	4	0
	Room 336	4	0	0	0	0
	Front corridor	4	0	0	0	4
	Central corridor	8	0	0	0	8
	Rear corridor	/	/	/	/	/
	Reference measuring point next to building 23A in the direction of building 33/37	20	0	0	0	0

**12.07.2021**	Sampling location	*A. fumigatus*	*A. niger*	*A. flavus*	Mucorales	Other moulds
	Room 306	4	0	0	0	8
	Room 308	60	8	0	0	4
	Room 310	8	0	0	0	12
	Room 312	96	4	0	0	8
	Room 314	32	8	0	0	8
	Room 316	56	0	0	0	8
	Room 318	76	8	0	0	0
	Room 322	44	8	0	4	4
	Room 324	36	4	0	4	8
	Room 326	12	0	0	0	0
	Room 328	0	4	0	4	0
	Room 330	4	0	0	0	0
	Room 332	8	0	0	0	0
	Room 334	28	0	0	0	0
	Room 336	0	0	0	0	0
	Front corridor	24	4	4	0	4
	Central corridor	56	0	0	0	4
	Rear corridor	8	0	0	0	0
	Reference measuring point next to building 23A in the direction of building 33/37	20	0	0	0	12
**18.08.2021**	Sampling location	*A. fumigatus*	*A. niger*	*A. flavus*	Mucorales	Other moulds
	Room 306	0	4	0	0	28
	Room 308	0	0	0	0	8
	Room 310	0	0	0	0	12
	Room 312	4	0	0	0	20
	Room 314	4	4	0	0	28
	Room 316	16	0	0	0	24
	Room 318	4	0	0	0	0
	Room 322	4	0	0	0	12
	Room 324	0	0	0	0	12
	Room 326	0	4	0	0	20
	Room 328	4	0	0	0	8
	Room 330	0	0	0	0	4
	Room 332	8	0	0	0	16
	Room 334	4	0	0	0	16
	Room 336	4	0	0	0	4
	Front corridor	0	0	0	0	44
	Central corridor	4	0	0	0	8
	Rear corridor	8	0	0	0	4
	Reference measuring point next to building 23A in the direction of building 33/37	24	8	0	0	20

**20.09.2021**	Sampling location	*A. fumigatus*	*A. niger*	*A. flavus*	Mucorales	Other moulds
	Room 306	28	4	0	0	8
	Room 308	24	4	0	0	0
	Room 310	4	4	0	0	0
	Room 312	4	0	0	0	4
	Room 314	0	0	0	0	0
	Room 316	12	0	0	0	0
	Room 318	4	0	0	0	0
	Room 322	0	0	0	0	0
	Room 324	12	0	0	0	4
	Room 326	8	0	0	0	4
	Room 328	8	0	0	0	0
	Room 330	0	0	0	0	0
	Room 332	0	0	0	0	0
	Room 334	0	0	0	0	0
	Room 336	0	4	0	0	4
	Front corridor	40	4	0	0	0
	Central corridor	8	0	0	0	4
	Rear corridor	8	0	0	0	4
	Reference measuring point next to building 23A in the direction of building 33/37	48	0	0	0	12
**18.10.2021**	Sampling location	*A. fumigatus*	*A. niger*	*A. flavus*	Mucorales	Other moulds
	Room 306	4	0	0	0	8
	Room 308	4	0	0	0	0
	Room 310	0	0	0	0	0
	Room 312	0	0	0	0	0
	Room 314	4	0	0	0	4
	Room 316	0	0	0	0	0
	Room 318	4	0	0	0	4
	Room 322	4	0	0	0	0
	Room 324	0	0	0	0	0
	Room 326	0	0	0	0	0
	Room 328	4	0	0	0	0
	Room 330	0	0	0	0	4
	Room 332	0	0	0	0	4
	Room 334	0	0	0	0	0
	Room 336	0	0	0	0	0
	Front corridor	4	4	0	0	8
	Central corridor	0	0	0	0	0
	Rear corridor	0	0	0	0	0
	Reference measuring point next to building 23A in the direction of building 33/37	8	0	0	0	12

**15.11.2021**	Sampling location	*A. fumigatus*	*A. niger*	*A. flavus*	Mucorales	Other moulds
	Room 306	0	0	0	0	0
	Room 308	0	0	0	0	0
	Room 310	0	0	0	0	0
	Room 312	4	0	0	0	4
	Room 314	0	0	0	0	0
	Room 316	0	0	0	0	0
	Room 318	12	0	8	0	0
	Room 322	0	0	0	0	0
	Room 324	0	0	0	0	0
	Room 326	4	4	0	0	4
	Room 328	0	0	0	0	0
	Room 330	0	0	0	0	0
	Room 332	0	0	0	0	0
	Room 334	0	0	0	0	0
	Room 336	0	0	0	0	0
	Front corridor	4	0	0	0	4
	Central corridor	4	0	12	0	0
	Rear corridor	4	0	0	0	0
	Reference measuring point next to building 23A in the direction of building 33/37	8	4	0	0	4
**14.12.2021****	Sampling location	*A. fumigatus*	*A. niger*	*A. flavus*	Mucorales	Other moulds
	Room B17	0	0	0	0	0
	Room B12	0	0	0	0	4
	Room B11	4	4	0	0	8
	Room B10	0	0	0	0	4
	Room B9	0	0	0	0	0
	Room B8	0	4	0	0	0
	Room B7	4	0	0	0	4
	Room B6	8	0	0	0	0
	Room B5	0	0	0	0	0
	Room B4	0	0	0	0	0
	Room B3	8	0	0	0	0
	Front corridor	0	0	0	0	0
	Central corridor	0	0	0	0	0
	Rear corridor	4	0	0	0	0
	Reference measuring point in front of building 23-clinic side (direction of building 15)	12	4	0	0	4
	Reference measuring point in front of building 23-Main side (in front of the main entrance)	8	4	0	0	0

**ward relocation from ward A0 (building 23A) to ward 10B (building 23B)

Table S10: Mould values (CFU/m^3), raw data 2022, ward A0, UHF.

	Sampling location	*A. fumigatus*	*A. niger*	*A. flavus*	Mucorales	Other moulds
**17.01.2022****	Sampling location	*A. fumigatus*	*A. niger*	*A. flavus*	Mucorales	Other moulds
	Room B17	4	0	0	0	8
	Room B12	0	0	0	0	4
	Room B11	0	0	0	0	0
	Room B10	0	0	0	0	0
	Room B9	8	0	0	0	0
	Room B8	0	0	0	0	4
	Room B7	4	0	0	0	4
	Room B6	0	0	0	0	0
	Room B5	12	0	0	0	0
	Room B4	0	0	0	0	0
	Room B3	0	0	0	0	0
	Front corridor	0	0	0	0	0
	Central corridor	4	0	0	0	0
	Rear corridor	0	0	0	0	0
	Reference measuring point in front of building 23-clinic side (direction of building 15)	8	0	0	0	4
	Reference measuring point in front of building 23-Main side (in front of the main entrance)	4	0	0	0	8
**14.02.2022****	Sampling location	*A. fumigatus*	*A. niger*	*A. flavus*	Mucorales	Other moulds
	Room B17	8	0	0	0	0
	Room B12	0	4	0	0	0
	Room B11	20	0	0	0	0
	Room B10	4	4	0	0	0
	Room B9	4	0	0	0	0
	Room B8	8	0	0	0	0
	Room B7	16	4	0	0	16
	Room B6	4	0	0	0	8
	Room B5	4	0	0	0	0
	Room B4	12	0	0	0	0
	Room B3	4	0	0	0	0
	Front corridor	4	0	0	0	0
	Central corridor	4	0	0	0	0
	Rear corridor	8	0	0	0	0
	Reference measuring point in front of building 23-clinic side (direction of building 15)	8	0	0	0	0
	Reference measuring point in front of building 23-Main side (in front of the main entrance)	20	0	0	0	4

**07.03.2022**	Sampling location	*A. fumigatus*	*A. niger*	*A. flavus*	Mucorales	Other moulds
	Room 306	12	0	0	0	0
	Room 308	0	0	0	0	4
	Room 310	0	0	0	0	0
	Room 312	4	0	0	0	0
	Room 314	0	0	0	0	0
	Room 316	0	0	0	0	0
	Room 318	0	0	0	0	0
	Room 322	0	0	0	0	0
	Room 324	0	0	0	0	0
	Room 326	0	0	0	0	0
	Room 328	4	0	0	0	0
	Room 330	0	0	0	0	0
	Room 332	0	0	0	0	0
	Room 334	0	0	0	0	0
	Room 336	0	0	0	0	0
	Front corridor	0	0	0	0	0
	Central corridor	0	0	0	0	0
	Rear corridor	0	0	0	0	0
	Reference measuring point next to building 23A in the direction of building 33/37	0	0	0	0	0
**28.03.2022**	Sampling location	*A. fumigatus*	*A. niger*	*A. flavus*	Mucorales	Other moulds
	Room 306	16	0	0	0	0
	Room 308	0	0	0	0	0
	Room 310	0	0	0	0	0
	Room 312	16	0	0	0	4
	Room 314	8	4	0	0	8
	Room 316	8	0	0	0	0
	Room 318	4	0	0	0	0
	Room 322	8	0	0	0	0
	Room 324	0	0	0	0	0
	Room 326	0	0	0	0	0
	Room 328	12	0	0	0	8
	Room 330	12	0	0	0	0
	Room 332	12	0	0	4	0
	Room 334	0	0	0	0	0
	Room 336	0	0	0	0	4
	Front corridor	0	0	0	0	4
	Central corridor	4	0	0	0	4
	Rear corridor	4	4	0	0	16
	Reference measuring point next to building 23A in the direction of building 33/37	0	0	0	0	0

**20.04.2022**	Sampling location	*A. fumigatus*	*A. niger*	*A. flavus*	Mucorales	Other moulds
	Room 306	0	0	0	0	0
	Room 308	8	0	0	0	0
	Room 310	8	0	0	0	0
	Room 312	8	0	0	0	12
	Room 314	24	0	0	0	16
	Room 316	4	4	0	0	12
	Room 318	8	0	0	0	8
	Room 322	24	4	0	0	4
	Room 324	4	0	0	0	0
	Room 326	4	0	0	0	4
	Room 328	8	0	0	0	0
	Room 330	0	0	0	0	0
	Room 332	8	0	0	0	4
	Room 334	36	0	0	0	12
	Room 336	0	0	0	0	0
	Front corridor	12	0	0	0	16
	Central corridor	28	0	0	0	12
	Rear corridor	16	8	0	0	4
	Reference measuring point next to building 23A in the direction of building 33/37	136	4	0	0	12

**16.05.2022**	Sampling location	*A. fumigatus*	*A. niger*	*A. flavus*	Mucorales	Other moulds
	Room 306	0	0	0	0	0
	Room 308	4	0	0	0	0
	Room 310	0	0	0	0	0
	Room 312	0	0	0	0	0
	Room 314	0	0	0	0	0
	Room 316	4	12	0	0	0
	Room 318	4	0	0	0	0
	Room 322	4	0	0	0	0
	Room 324	0	0	0	0	0
	Room 326	4	0	0	0	0
	Room 328	4	0	0	0	0
	Room 330	0	0	0	0	0
	Room 332	0	0	0	0	0
	Room 334	0	0	0	0	0
	Room 336	4	0	0	0	0
	Front corridor	8	0	0	0	0
	Central corridor	4	0	0	0	0
	Rear corridor	4	4	0	0	0
	Reference measuring point next to building 23A in the direction of building 33/37	8	4	0	0	0

**20.06.2022**	Sampling location	*A. fumigatus*	*A. niger*	*A. flavus*	Mucorales	Other moulds
	Room 306	0	0	0	0	0
	Room 308	4	0	0	0	4
	Room 310	4	0	0	0	0
	Room 312	12	0	0	0	0
	Room 314	28	4	0	0	0
	Room 316	28	4	0	0	0
	Room 318	8	0	0	0	4
	Room 322	12	4	0	0	4
	Room 324	4	0	0	0	0
	Room 326	32	4	0	4	12
	Room 328	44	0	0	0	8
	Room 330	20	0	0	0	12
	Room 332	4	0	0	0	0
	Room 334	12	4	0	0	0
	Room 336	12	0	0	0	4
	Front corridor	8	0	0	0	0
	Central corridor	28	0	0	0	0
	Rear corridor	12	4	0	0	8
	Reference measuring point next to building 23A in the direction of building 33/37	8	0	0	0	0
**18.07.2022**	Sampling location	*A. fumigatus*	*A. niger*	*A. flavus*	Mucorales	Other moulds
	Room 306	4	4	0	0	0
	Room 308	0	0	0	0	12
	Room 310	0	0	0	8	24
	Room 312	0	0	0	0	0
	Room 314	4	12	0	0	0
	Room 316	20	4	0	4	4
	Room 318	4	8	0	0	4
	Room 322	12	12	0	0	16
	Room 324	8	0	0	0	0
	Room 326	4	8	0	4	4
	Room 328	12	4	0	0	4
	Room 330	12	4	0	0	0
	Room 332	0	0	0	0	0
	Room 334	16	0	0	0	16
	Room 336	8	4	0	0	4
	Front corridor	8	12	4	0	4
	Central corridor	8	0	0	0	12
	Rear corridor	4	4	0	0	12
	Reference measuring point next to building 23A in the direction of building 33/37	0	0	0	4	40

	Sampling location	*A. fumigatus*	*A. niger*	*A. flavus*	Mucorales	Other moulds
**24.08.2022**	Sampling location	*A. fumigatus*	*A. niger*	*A. flavus*	Mucorales	Other moulds
	Room 306	0	0	0	0	0
	Room 308	8	8	0	4	0
	Room 310	8	4	0	0	4
	Room 312	8	4	0	4	4
	Room 314	4	28	0	4	16
	Room 316	8	20	0	4	28
	Room 318	12	12	0	4	4
	Room 322	20	8	4	0	16
	Room 324	24	8	0	0	12
	Room 326	8	32	8	0	36
	Room 328	24	12	0	0	4
	Room 330	24	24	0	0	28
	Room 332	/	/	/	/	/
	Room 334	0	24	0	4	16
	Room 336	4	0	0	0	0
	Front corridor	40	24	0	0	8
	Central corridor	28	20	0	4	12
	Rear corridor	4	4	0	0	4
	Reference measuring point next to building 23A in the direction of building 33/37	8	32	8	0	16
**19.09.2022**	Sampling location	*A. fumigatus*	*A. niger*	*A. flavus*	Mucorales	Other moulds
	Room 306	4	0	0	0	4
	Room 308	0	0	0	0	4
	Room 310	16	8	0	0	8
	Room 312	8	0	0	0	0
	Room 314	4	0	0	0	12
	Room 316	8	4	0	0	12
	Room 318	0	0	0	0	4
	Room 322	0	0	0	0	8
	Room 324	0	0	0	0	0
	Room 326	0	0	0	0	4
	Room 328	0	0	0	0	4
	Room 330	0	0	0	0	4
	Room 332	4	0	0	0	0
	Room 334	0	0	0	0	4
	Room 336	0	0	0	0	0
	Front corridor	0	0	0	0	16
	Central corridor	0	0	0	0	8
	Rear corridor	4	0	0	0	0
	Reference measuring point next to building 23A in the direction of building 33/37	16	0	0	0	4

**17.10.2022**	Sampling location	*A. fumigatus*	*A. niger*	*A. flavus*	Mucorales	Other moulds
	Room 306	8	0	0	4	0
	Room 308	24	8	0	4	4
	Room 310	8	0	0	0	8
	Room 312	20	4	0	0	12
	Room 314	8	0	0	4	4
	Room 316	24	8	0	0	16
	Room 318	0	4	0	0	28
	Room 322	20	0	0	0	16
	Room 324	16	0	0	0	8
	Room 326	8	8	8	0	8
	Room 328	0	0	0	0	8
	Room 330	0	4	0	0	8
	Room 332	/	/	/	/	/
	Room 334	/	/	/	/	/
	Room 336	/	/	/	/	/
	Front corridor	16	4	0	0	8
	Central corridor	20	4	4	0	12
	Rear corridor	20	8	8	0	8
	Reference measuring point next to building 23A in the direction of building 33/37	24	0	0	0	20
**14.11.2022**	Sampling location	*A. fumigatus*	*A. niger*	*A. flavus*	Mucorales	Other moulds
	Room 306	4	0	0	0	0
	Room 308	12	0	0	0	0
	Room 310	0	0	0	0	0
	Room 312	0	0	0	0	0
	Room 314	16	0	0	0	0
	Room 316	20	0	0	0	0
	Room 318	8	0	0	0	4
	Room 322	4	0	0	0	4
	Room 324	8	0	0	0	0
	Room 326	24	0	0	4	4
	Room 328	0	0	0	0	0
	Room 330	0	0	0	0	0
	Room 332	/	/	/	/	/
	Room 334	/	/	/	/	/
	Room 336	/	/	/	/	/
	Front corridor	0	0	0	4	0
	Central corridor	16	0	0	4	0
	Rear corridor	8	4	0	0	0
	Reference measuring point next to building 23A in the direction of building 33/37	28	4	0	0	8

**19.12.2022**	Sampling location	*A. fumigatus*	*A. niger*	*A. flavus*	Mucorales	Other moulds
	Room 306	0	0	0	0	0
	Room 308	0	0	0	0	0
	Room 310	0	0	0	0	0
	Room 312	0	0	0	0	0
	Room 314	0	0	0	0	0
	Room 316	0	0	0	0	0
	Room 318	0	0	0	0	4
	Room 322	0	0	0	0	0
	Room 324	0	0	0	0	0
	Room 326	0	0	0	0	0
	Room 328	0	0	0	0	0
	Room 330	0	0	0	0	0
	Room 332	0	0	0	0	0
	Room 334	0	0	0	0	0
	Room 336	0	0	0	0	0
	Front corridor	4	0	0	0	0
	Central corridor	8	0	0	0	0
	Rear corridor	0	0	0	0	0
	Reference measuring point next to building 23A in the direction of building 33/37	4	0	0	0	4

**ward relocation from ward A0 (building 23A) to ward 10B (building 23B)

Table S11: CDC weather data for weather station ID 1420 (2018).

2018/ Month	air temperature [°C]	relative humidity [%]
I	/	/
II	2	87
III	-2	51
IV	10	92
V	16	45
VI	23	66
VII	21	66
VIII (I)	30	40
VIII (II)	17	55
IX	12	69
X	/	/
XI	10	75
XII	/	/

(I)beginning of the month; (II)end of the month

Table S12: CDC weather data for weather station ID 1420 (2019).

2019/ Month	air temperature [°C]	relative humidity [%]
I	/	/
II	2	77
III	5	73
IV	/	/
V	12	57
VI	15	76
VII	/	/
VIII (I)	20	70
VIII (II)	23	57
IX	/	/
X	12	87
XI	7	80
XII	5	95

(I)beginning of the month; (II)end of the month

Table S13: CDC weather data for weather station ID 1420 (2020).

2020/ Month	air temperature [°C]	relative humidity [%]
I (I)	8	69
I (II)	13	80
II	5	70
III	/	/
IV*	13	45
V*	17	49
VI	12	76
VII (I)	19	57
VII (II)	21	50
VIII	27	42
IX	21	58
X	9	80
XI	4	83
XII	1	98

(I)beginning of the month; (II)end of the month; * ward relocation from ward A0 (building 23A) to ward 28-1 (building 28)

Table S14: CDC weather data for weather station ID 1420 (2021).

2021/ Month	air temperature [°C]	relative humidity [%]
I	2	78
II	-1	70
III	6	79
IV	9	81
V	12	76
VI	20	53
VII	21	73
VIII	18	77
IX	14	66
X	8	90
XI	8	78
XII**	6	93

(I)beginning of the month; (II)end of the month; ** ward relocation from ward A0 (building 23A) to ward 10B (building 23B)

Table S15: CDC weather data for weather station ID 1420 (2022).

2022/ Month	air temperature [°C]	relative humidity [%]
**I****	5	80
**II****	6	77
**III (I)**	2	48
**III (II)**	13	55
**IV**	12	46
**V**	19	67
**VI**	17	67
**VII**	25	32
**VIII**	26	40
**IX**	12	72
**X**	17	86
**XI**	7	89
**XII**	-1	92

(I)beginning of the month; (II)end of the month; ** ward relocation from ward A0 (building 23A) to ward 10B (building 23B)
